# Infectious Complications Are Associated With Alterations in the Gut Microbiome in Pediatric Patients With Acute Lymphoblastic Leukemia

**DOI:** 10.3389/fcimb.2019.00028

**Published:** 2019-02-19

**Authors:** Jacob T. Nearing, Jessica Connors, Scott Whitehouse, Johan Van Limbergen, Tamara Macdonald, Ketan Kulkarni, Morgan G. I. Langille

**Affiliations:** ^1^Department of Microbiology and Immunology, Dalhousie University, Halifax, NS, Canada; ^2^Division of Gastroenterology, Department of Pediatrics, IWK Health Centre, Halifax, NS, Canada; ^3^Pediatric Gastroenterology and Nutrition, Emma Children's Hospital, Amsterdam University Medical Center, Amsterdam, Netherlands; ^4^Division of Hematology/Oncology, Department of Pediatrics, IWK Health Centre, Halifax, NS, Canada; ^5^Department of Pharmacology, Dalhousie University, Halifax, NS, Canada

**Keywords:** microbiome, genomics, cancer, leukemia, clinical, infection, metagenomics

## Abstract

Acute lymphoblastic leukemia is the most common pediatric cancer. Fortunately, survival rates exceed 90%, however, infectious complications remain a significant issue that can cause reductions in the quality of life and prognosis of patients. Recently, numerous studies have linked shifts in the gut microbiome composition to infection events in various hematological malignances including acute lymphoblastic leukemia (ALL). These studies have been limited to observing broad taxonomic changes using 16S rRNA gene profiling, while missing possible differences within microbial functions encoded by individual species. In this study we present the first combined 16S rRNA gene and metagenomic shotgun sequencing study on the gut microbiome of an independent pediatric ALL cohort during treatment. In this study we found distinctive differences in alpha diversity and beta diversity in samples from patients with infectious complications in the first 6 months of therapy. We were also able to find specific species and functional pathways that were significantly different in relative abundance between samples that came from patients with infectious complications. Finally, machine learning models based on patient metadata and bacterial species were able to classify samples with high accuracy (84.09%), with bacterial species being the most important classifying features. This study strengthens our understanding of the association between infection and pediatric acute lymphoblastic leukemia treatment and warrants further investigation in the future.

## Introduction

Acute lymphoblastic leukemia (ALL) is the most common pediatric cancer (Canadian Cancer and Statistics Advisory Committee, 2018). Recently, numerous studies have linked the gut microbiome to affecting treatment outcomes and infection status in multiple different types of cancers such as acute myeloblastic leukemia (Galloway-Peña et al., 2016), non-Hodgkin's lymphoma (Montassier et al., 2016), hematopoietic stem cell transplant patients (Taur et al., 2014), as well as acute lymphoblastic leukemia (Hakim et al., 2018). In all of these clinical scenarios, treatment involves harsh chemotherapeutics that can cause disruption of the structural integrity of the gut barrier, which has been proposed to allow the passage of gut bacteria into the blood and throughout the body (Berg, 1995). The gut microbiome and the immune system also share an intimate relationship, as disruptions in the microbial community have been linked to inflammation and reduced immune function (Belkaid and Hand, 2014).

Earlier studies on the gut microbiome and pediatric acute lymphoblastic leukemia have found significant differences between individuals with ALL and healthy controls at base line (Rajagopala et al., 2016; Bai et al., 2017). Bai et al. found significant differences in alpha diversity between ALL patients that were not exposed to antibiotics and healthy controls as well as differences in beta diversity regardless of antibiotic use. Similar findings were presented earlier by Rajagopala et al. where they were able to classify baseline samples as either coming from an individual diagnosed with ALL or a healthy sibling control based on their gut microbiome composition.

Building on this classification ability, it has been suggested that the gut microbiome could be used to predict infection in pediatric acute lymphoblastic leukemia patients. Hakim et al., showed that in a cohort of patients treated at the St. Jude Children's Research Hospital compositional signatures of the gut microbiome could be used to predict infections during treatment (Hakim et al., 2018). More specifically they found that a higher abundance of Proteobacteria at baseline was predictive of febrile neutropenia and that domination of the gut by either Enterococcaceae or Streptococcaceae increased the risk of infection throughout therapy. Possible differences in microbial functions and species within this cohort were not identified due to technical restrictions from using only 16S rRNA gene sequencing

Clear microbial associations between the gut microbiome and treatment complications remain to be discovered. We hypothesize that changes in the composition of gut microbial communities are associated with infectious complications during chemotherapy treatment. Herein, we present the first study to examine the gut microbial communities of pediatric acute lymphoblastic leukemia throughout the first 6 months of chemotherapy using both 16S rRNA gene sequencing and whole metagenomic shotgun sequencing. Through this examination we identify multiple significant differences in both taxonomic and functional profiles between samples from pediatric ALL patients with and without infectious complications throughout the first 6 months of therapy.

## Methods

### Patient Demographics

A total of 44 samples were collected from 16 patients treated at the IWK Health Center in Halifax, Nova Scotia, Canada. The ages of participants ranged from (0.75–11.12 years of age), with a mean age of 40.78 months and a total of 11 males and 5 females (Table 1). All females in the study suffered an infectious complication within the first 6 months of treatment. All samples were collected within 6 months of therapy with the exception of one baseline sample that was collected 2 days prior to the start of treatment (Supplementary Figure 1). The median number of samples collected from each patient was 2 with a range between 1 and 7 (Supplementary Figure 1). Nine patients faced an infectious complication within the first 6 months of treatment and 19 samples were collected from them in total. The other seven patients did not face infectious complications within the first 6 months of treatment and 25 samples were collected from them in total (Table 1). Baseline samples were classified as the first sample from a patient within the first 10 days of the start of therapy.

**Table 1:** Patient and sample data divided between age group, sex, and whether the patient did (IC) or did not have infectious complication (NIC) within the first 6 months of treatment.

**Diagnosis**	**Acute lymphoblastic leukemia: total patients = 16, Samples = 44**		
**Age group**	**Ages 0–4**	**Ages 5–8**	**Ages 9–11**
	**Patients = 9, samples = 27**	**Patients = 4, samples = 7**	**Patients = 3, samples = 10**
Male	Patients = 5, samples = 17	Patients = 4, samples = 7	Patients = 2, samples = 8
Male IC	Patients = 1, samples = 3	Patients = 2, samples = 2	Patients = 1, samples = 2
Male NIC	Patients = 4, samples = 14	Patients = 2, samples = 5	Patients = 1, samples = 6
Female	Patients = 4, samples = 10	Patients = 0, samples = 0	Patients = 1, samples = 2
Female IC	Patients = 4, samples = 10	Patients = 0, samples = 0	Patients = 1, samples = 2
Female NIC	Patients = 0, samples = 0	Patients = 0, samples = 0	Patients = 0, samples = 0

All patients/guardians provided informed written consent to participate in this study. Clinical data and stool samples were collected in accordance with protocols approved by the IWK Health Center Research Ethics Board (REB).

Infectious complications (IC) were defined as any microbially or clinically-defined infection and/or clinically-documented febrile neutropenia event. Samples from IC patients were later stratified into groups that were taken after the initial infectious complication (post) and before the initial infectious complication (pre). Samples were further classified based on whether the sample was taken before a blood stream infection event, gastrointestinal infection event or febrile neutropenia event.

Antibiotic exposure was defined as the administration of intravenous or oral antibiotics within 2 weeks prior to sample collection, excluding prophylactic Septra which was prescribed ubiquitously to patients throughout the study, as per standard of care. Antibiotic exposure was further categorized into exposure to anti-fungal medications (such as pentamidine and caspofungin), vancomycin, piperacillin tazobactam, and other uncommonly used antibiotics such as metronidazole and ceftazidime.

### Fecal Sample Collection

Stool samples were frozen at −20°C for transport to the laboratory and stored at −80°C until use. Total DNA was purified from each sample using the Norgen Stool DNA Isolation Kit (cat#27600, Norgen Biotek, Canada).

### 16S rRNA Gene Sequencing

16S rRNA gene sequencing was performed by the Integrated Microbiome Resource at Dalhousie University. Briefly, the V4-V5 16S rRNA gene region was amplified using the high-fidelity Phusion polymerase (cat#M0530L, New England Biolabs) and 16S rRNA V4-V5 fusion primers (Comeau et al., 2017). The amplified 16S V4-V5 regions were then sequenced using an Illumina MiSeq producing 300 bp paired-end reads. Of the total of 44 samples, 41 were sequenced via 16S rRNA amplicon sequencing, and all 44 were sequenced using shotgun metagenomics. Of the 41 samples sequenced via 16S rRNA amplicon sequencing 4 were removed by quality filters leaving a total of 37 samples. The three samples only sequenced by metagenomic shotgun sequencing was due to loss of the sample. This led to a total of 11 baseline samples being sequenced by 16S rRNA gene sequencing.

### Metagenomic Shotgun Sequencing

Metagenomic shotgun sequencing was performed by the Integrated Microbiome Resource at Dalhousie University. Briefly, extracted DNA was prepared into a sequencing library using the Illumina Nextera XT kit (cat#FC-131-1096, Illumina, USA). The prepared sequencing library was then sequenced on an Illumina Nextseq 550 producing 150 base pair paired end reads with a median read depth of (3,372,240) per sample. A total of 13 baseline samples were sequenced by metagenomic shotgun sequencing.

### 16S rRNA Sequence Analysis

Sequences were processed using QIIME2 (Bolyen et al., 2018) and the Deblur plugin (Amir et al., 2017). Paired end sequences were stitched together using the Microbiome Helper script (Comeau et al., 2017) run_pear.pl, which wraps PEAR (Zhang et al., 2014). Paired sequences were then imported into a QIIME2 artifact and filtered based on read quality and length using QIIME2's built in quality-filter q-score-joined script. Filtered reads were then processed with Deblur using a trim length of 300 base pairs to obtain amplicon sequence variants. In total 1,058 different amplicon sequence variants were observed over a median read depth of 6,527 reads and 37 samples. In order to acquire diversity metrics, the samples were subsampled to 1,481 reads per sample. Amplicon sequence variants were then analyzed using multiple alpha diversity metrics (shannon, evenness, observed-ASVs, faith's phylogenetic diversity) and statistical significance between samples from IC and NIC patients and other metadata variables such as age at diagnosis were determined using a Wilcoxon Rank Sum test. A classification model to determine samples from NIC or IC patients was created using a logistic regression model built in R (R Development Core Team, 2008) with faith's phylogenetic diversity, days since the start of therapy, treatment type, and age at diagnosis as predictors. Weighted UniFrac distances and Principal Coordination of Analysis ordination were generated using QIIME2 and the APE R package (Paradis and Schliep, 2018). Differences between samples weighted UniFrac distances were tested using the adonis2 function (PERMANOVA test) from the vegan R package (Oksanen et al., 2018). Taxonomy was assigned to ASVs using the rdp-classifier (Cole et al., 2014) trained on the Greengenes 13_8 database (DeSantis et al., 2006).

### Metagenomic Sequence Analysis

Metagenomic sequences were processed using the Microbiome Helper operating procedures for metagenomic data. In brief, contaminant reads were removed by mapping sequences to the human and PhiX genomes using bowtie2 (Langmead and Salzberg, 2012). The resulting reads were then classified taxonomically using MetaPhlAn2 (Truong et al., 2015) and into Metacyc pathways using HUMAnN2 (Franzosa et al., 2018). Metacyc pathways and taxa that were significantly different in abundance between NIC and IC patients and other metadata variables presented in the study were determined using a Wilcoxon rank sum test and corrected for false discovering using Benjamini & Hochberg correction.

HUMAnN2 species stratified pathway output was then filtered to only contain the pathways found to be significantly different. Pathways that had a total contribution from all species of <0.001% were removed. Species were then collapsed to their respective genera for visualization purposes with any genera that contributed to <0.01% of the total relative abundance of the significant pathways being grouped into “Other.”

To determine the prevalence of antibiotic resistance genes and virulence factor genes we aligned reads against the Comprehensive Antibiotic Resistance Database (CARD) (Jia et al., 2017) and the Virulence Factor Databases (VFDB) respectively, (Chen et al., 2005) using DIAMOND with a default cutoff e value of 0.001 and a maximum number of one target sequence (Buchfink et al., 2014). Differences between samples from NIC and IC patients in prevalence of antimicrobial resistance genes and virulence factors were tested using a Wilcoxon rank sum test. Differential abundant antimicrobial resistance genes and virulence factor genes were determined using a Wilcoxon rank sum test with correction for false discovering using Benjamini & Hochberg correction.

To get a sense of the importance of various features for classification of samples we employed the use of random forest modeling. The MetaPhlAn2 species table was combined with metadata on the same samples and used as the features for sample classification. A random forest model was then constructed in R using the randomForest package (Liaw and Wiener, 2002) with default hyperparameters and 10,001 trees. The accuracy of the model was determined by examining the out of bag error and feature importance was determined by looking at the mean decrease in accuracy for each classification feature.

## Results

### Microbiome Profiling Reveals Difference in Phylogenetic Diversity Between NIC and IC Patients, Subsequent Blood Stream Infections, Age and Time of Treatment

**Figure 1:**
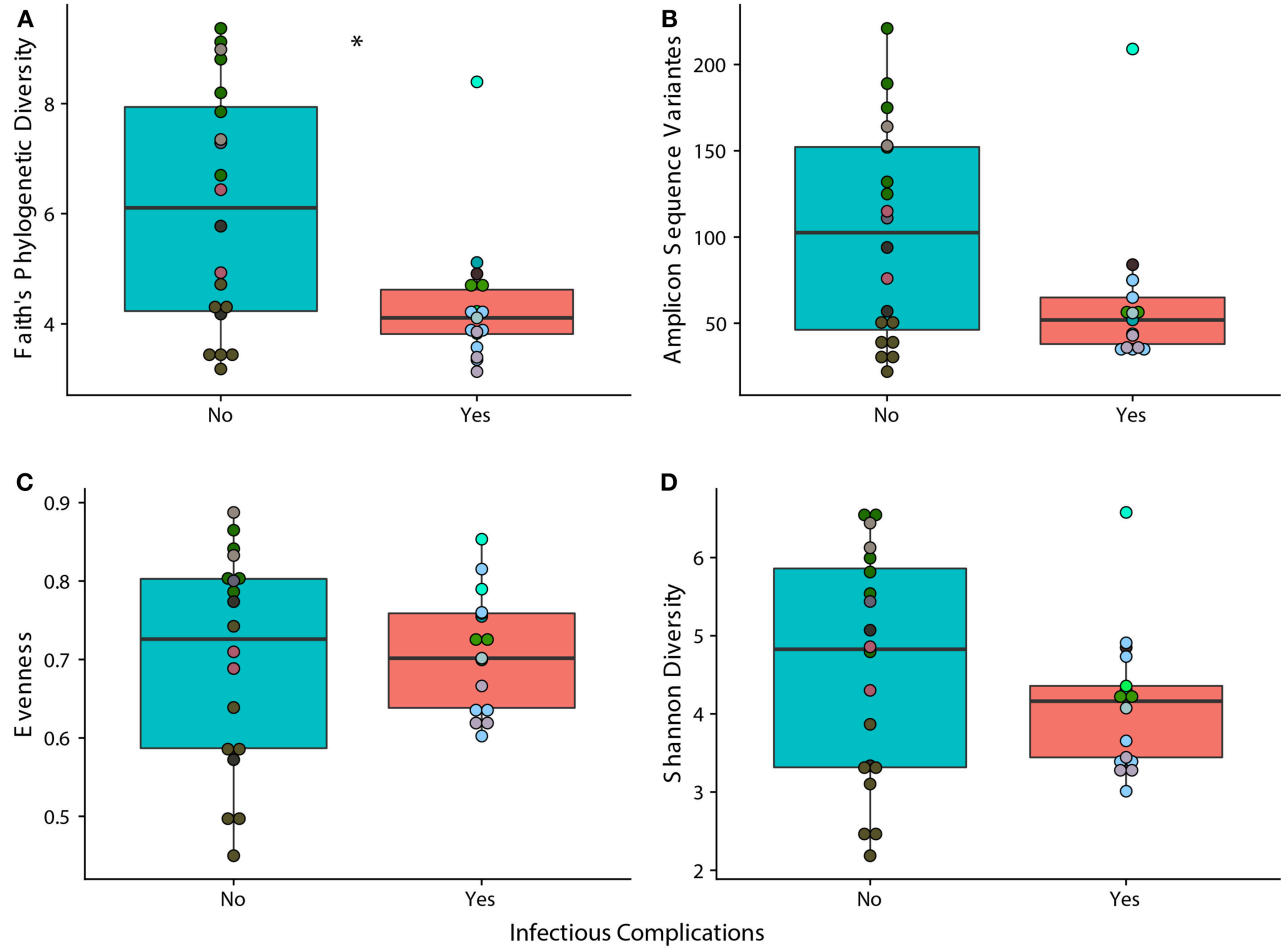
Phylogenetic Diversity based on 16S rRNA gene sequencing is significantly different between samples from patients that face infectious complications (IC) within the first 6 months of therapy and those that do not (NIC). Differences in alpha diversity between samples from IC and NIC patients that were sequences by 16S rRNA gene sequencing at a read depth of 1481 (37 of 44 total samples). Significance was determined using a Wilcoxon rank sum test at an alpha value of 0.05 (represented by ^*^). Each panel represents a different measure of alpha diversity; shannon diversity **(A)**, number of amplicon sequence variants **(B)**, evenness **(C)**, and Faith's phylogenetic diversity **(D)**. Points are colored by individual (Supplementary Figure 1).

We observed that samples that were taken from patients that experienced an infectious complication within the first 6 months of therapy (IC) had lower phylogenetic diversity when compared to samples taken from patients that did not face an infectious complication within 6 months of therapy (NIC). (Figure 1A; *p* = 0.014). However, measures in richness and evenness were similar between samples from IC and NIC patients (Figures 1B–D). To determine if a specific type of infection was related to this difference in phylogenetic diversity, we looked at how subsequent bloodstream and gastrointestinal infections were related to phylogenetic diversity. Interestingly, we did find that a lower Faith's phylogenetic diversity was significantly associated with subsequent bloodstream infections (*p* = 0.0469) (Supplementary Figure 2). Following this analysis, we were interested in looking at what other factors may be impacting phylogenetic diversity in our patient cohort.

As it is well-known that the composition of the gut microbiome changes with age, we examined how age at diagnosis impacted phylogenetic diversity. This resulted in a significant positive correlation (*p* = 0.0001, rho = 0.656) indicating that age could be playing a role in the differences we see between samples from NIC and IC patients (Supplementary Figure 3). Examining changes in alpha diversity over time on a per patient basis was not possible due to highly variable sampling periods and therefore we opted to look at overall changes in alpha diversity over time. We found that the number of days after the start of treatment was also significantly negatively correlated with Faith's Phylogenetic Diversity (*p* = 0.01327, rho = −0.403) indicating that chemotherapy treatment may reduce overall gut diversity (Supplementary Figure 4).

To test classification accuracies of samples coming from NIC or IC patients while accounting for the above confounding factors we built a logistic regression model based on Faith's phylogenetic diversity, treatment type, sample collection time, and age at diagnosis. Our model was able to classify samples that were from IC or NIC patients with an area under the curve of 0.77, and a kappa value of 0.46 (Supplementary Figure 5). No single predictor in the model was significant however, Faith's phylogenetic diversity was near our significant alpha value (*p* = 0.0994). We could not build a model that accounted for differences in sex due to all females being classified as IC patients (Table 1). We, however, did not find a significant association between sex and Faith's phylogenetic diversity (*p* = 0.2149).

Inspecting other possible confounding factors lead us to looking at the inevitably close relationship between the use of antibiotics and infection events (due to strict adherence to febrile neutropenia treatment protocols). Due to this relationship we decided to determine how individual antibiotic exposures were related to phylogenetic diversity of the gut microbiome. We observed a significant decrease in phylogenetic diversity and the usage of vancomycin (Wilcoxon rank sum test; *p* = 0.039), while there were no significant associations with the usage of piperacillin-tazobactam, antifungal medications, or any other antibiotics including total antibiotic exposure (Supplementary Figure 6). Furthermore, we found that all antibiotics except for piperacillin-tazobactam were highly associated with infectious complications (Fisher's Exact test; OR = 6.03, *p* = 0.0323), however, they were not significantly associated to phylogenetic diversity (Wilcoxon rank sum test; *p* = 0.2534). Despite this result it is difficult to tell the full extent of the impact that antibiotic usage has on the gut microbiome of patients within the cohort due to our small sampling size and sparse usage of some medications such as anti-fungal treatment.

Following our analysis on antibiotic exposure, we were also interested in examining the differences between samples that came from patients that never experience infectious complications and samples that were pre or post their first infectious complication event. We found no significant differences between samples that were pre or post-infection, or pre-infection and samples from patients that never experience infectious complications (Supplementary Figure 7). We did, however, find significant differences in phylogenetic diversity between post infection samples and samples that came from NIC patients (*p* = 0.0113) (Supplementary Figure 7). Antibiotic exposure to antibiotics other than piperacillin tazobactam was not significantly associated with changes in phylogenetic diversity between samples that came from patients that never faced infection and post infection samples (*p* = 0.358). In addition, no individual antibiotic exposure was significantly associated, however, vancomycin exposure was close to our alpha value (*p* = 0.1249).

Finally, we examined 11 baseline samples (Supplementary Figure 1) (taken within 10 days of the start of therapy) to determine whether alpha diversity at baseline was predictive of future infections and minimal residual disease (MRD) at 30 days. We did not find a significant association between Faith's phylogenetic diversity and NIC or IC status (Wilcoxon rank sum test; *p* = 0.1255). Stratifying the infection types into either bloodstream infections, gastrointestinal infections, or febrile neutropenia did reveal a significant association between baseline alpha diversity and subsequent bloodstream infections (Wilcoxon rank sum test; *p* = 0.02424). We did not find a significant relationship between MRD at 30 days and baseline Faith's Phylogenetic Diversity (Wilcoxon rank sum test; *p* = 0.6303).

### Microbiome Profiling Reveals Significant Differences in Beta Diversity Between Samples From NIC and IC Patients

**Figure 2:**
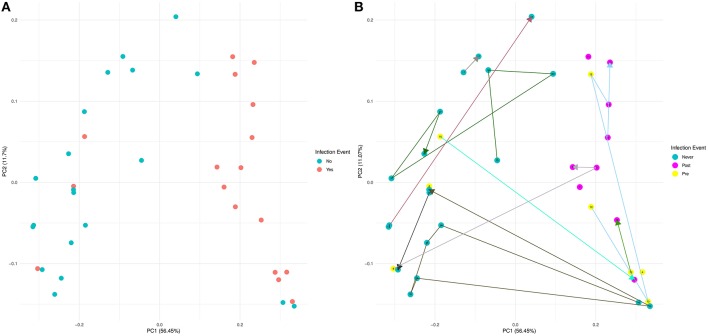
Weighted UniFrac beta diversity (16S rRNA gene sequencing) is significantly different between samples from NIC and IC patients. A Principal Coordinates of Analysis ordination plot of the weighted UniFrac distances of the samples sequenced by 16S rRNA gene sequencing at a read depth of 1481 (37 of 44 samples). **(A)** Samples that came from NIC patients are colored blue and samples from IC patients are colored red. **(B)** Samples colored based on whether a sample came from an NIC patient (never in blue) or an IC patient pre (yellow) or post (purple) their initial infectious complication. Lines connecting samples represent the chronological order of sample collection from each patient. Numbers on points and the color connecting points together are colored by individual (Supplementary Figure 1).

Using 16S rRNA gene sequencing and weighted UniFrac analysis to account for both abundance and phylogenetic distance, we found significant differences between samples from IC and NIC patients (PERMANOVA, *r*^2^ = 0.2112, *p* = 0.001). Plotting weighted UniFrac distances using principal coordinates of analysis (PCoA) ordination resulted in distinct separations between samples from NIC and IC patients (Figure 2A). To test what other factors may be associated with the differences between samples from NIC and IC patients we completed both univariate and multivariate analyses using PERMANOVA tests. Along with differences between IC and NIC samples we also found differences in sex (although all females are IC patients), days since the start of therapy (Supplementary Figure 8), vancomycin exposure and anti- fungal exposure (Table 2). We did not find significant differences in microbial community structure in patients based on age at diagnosis (Supplementary Figure 8), treatment type, overall antibiotic exposure, piperacillin tazobactam exposure, or other less commonly used antibiotics (Table 2). Following this we tested all features significantly associated with changes in weighted UniFrac within a single multivariate PERMANOVA test to try and determine the most important features. We found that none of the factors alone were significant explanatory variables, however, infectious complications explained the most variance among all the predictors (*R*^2^ = 0.04433, *p* = 0.08) (Table 3). In order to get a better visualization of how samples with antifungal or vancomycin exposure compared to those that did not, we recolored our PCoA based of these two exposure outcomes (Supplementary Figure 9). From this we noted that not all samples from IC patients that grouped within the IC cluster were exposed to vancomycin or antifungal medication. We also found that three of the five samples that were exposed to anti-fungal medication came from one patient, which could be playing a role in the significant association that we found.

**Table 2:** Univariate weighted UniFrac analysis on multiple metadata features using a single PERMANOVA test for each metadata feature.

**Feature**	***R*^**2**^**	***P***
**UNIVARIATE ANALYSIS**		
Infection in 6 months	0.2112	0.0003
Sex	0.15456	0.0024
Age at diagnosis	0.04421	0.159
Within 2 weeks of antibiotic exposure (not including Septra)	0.01716	0.606
Within 2 Weeks of Vancomyicin Exposure	0.12044	0.004
Within 2 weeks of anti-fungal exposure	0.09899	0.013
Within 2 weeks of Piperacillin Tazobactem exposure	0.00977	0.883
Within 2 weeks of other antibiotic exposure	0.02664	0.373
Days since start of therapy	0.08903	0.032
Treatment type	0.04676	0.1444

**Table 3:** Multivariate weighted UniFrac analysis on multiple metadata features using a single PERMANOVA test containing all of the features found to be significant in univariate analysis.

**Feature**	***R*^**2**^**	***P***
**SIGNIFICANT MULTIVARIATE ANALYSIS**		
Infection in 6 Months	0.04433	0.08
Sex	0.01939	0.392
Days since therapy start	0.03684	0.149
Within 2 weeks of vancomycin exposure	0.02021	0.392
Within 2 weeks of anti-fungal exposure	0.01979	0.423

Similar to our analysis with alpha diversity, we were interested in comparing samples from patients that never experience infection vs. pre and post initial infectious complication samples. Visualizing these results using a PCoA that connected samples from the same patient in chronological order revealed that samples from IC patients that previously grouped with NIC patients were pre-infection samples (Figure 2B). We tested whether any of these groups were significantly different from each other and found that samples from NIC patients grouped significantly from pre (*R*^2^ = 0.109, *p* = 0.027) and post (*R*^2^ = 0.294, *p* = 0.001) IC samples. We did not find a significant difference between pre and post IC samples (*R*^2^ = 0.1018, *p* = 0.149).

### NIC and IC Patients Differ in Several Bacterial Species

**Figure 3:**
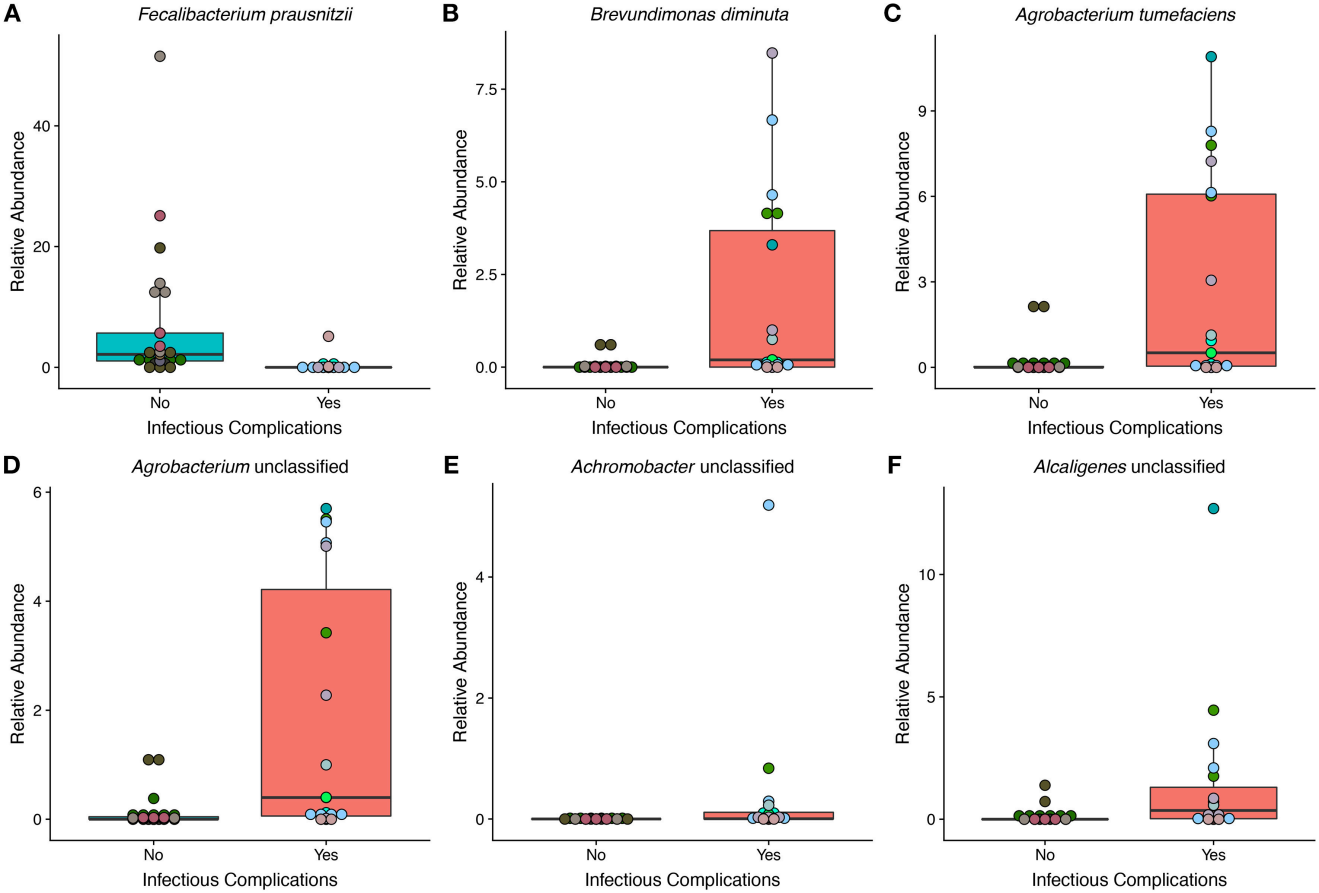
The relative abundance of multiple species is significantly different between samples from NIC and IC patients that were sequenced by metagenomic shotgun sequencing. Six species were found to be significantly different in relative abundance between samples from NIC and IC patients: *Fecalibacterium prausnitzii* **(A)**, *Brevundimonas diminuta* **(B)**, *Agrobacterium tumefaciens* **(C)**, *Agrobacterium unclassified* **(D)**, *Achromobacter unclassified* **(E)**, and *Alcaligenes unclassified* **(F)**. (Wilcoxon rank sum test with correction for false discovery at an alpha value of 0.05). Points are colored by individual patient (Supplementary Figure 1).

To get a high-resolution view of the gut microbial communities of patients enrolled in the study we looked at the taxonomic profiles generated from shotgun metagenomics. We found a total of 6 different species in differential abundance between NIC and IC patients using a Wilcoxon rank sum test and correction for false discovery (Figure 3). We found that the only bacterial species that was in higher relative abundance in samples from NIC patients was *Faecalibacterium prausnitzii*. Interestingly this species was almost completely absent in samples from IC patients, apart from 3 samples (Figure 3A). The rest of the species that we identified as being in differential abundance had higher relative abundances in samples from IC patients when compared to samples from NIC patients (Figures 3B–F). The mean difference in relative abundance between these species ranged from 0.36% to 6.5%. Furthermore, many of these species were not present in many samples. *Faecalibacterium prausnitzii* was the only species present in all the samples from either NIC or IC patients (Figure 3A).

We did a similar analysis at multiple taxonomic levels in order to assess the large collective shifts of microbial groups within the gut microbiome of samples from NIC and IC patients. We found multiple differentially abundant taxa at multiple taxonomic levels based on metagenomic shotgun sequencing. At the phylum level we found Bacteroidetes to be significantly enriched in relative abundance in samples from NIC patients and found Proteobacteria to be significantly reduced in NIC patients (Supplementary Figure 10). We found six families to be in significantly increased relative abundance in samples from NIC patients Bifidobacteriaceae, Bacteroidaceae, Prevotellaceae, Rikenellaceae, and Eubacteriaceae (Supplementary Figure 11). All four families that were in increased relative abundance in samples from IC patients were part of the Proteobacteria phylum (Caulobacteraceae, Rhizobiaceae, Alcaligenaceae, and Burkholderiaceae) (Supplementary Figure 11). At the genus level, we found similar results with six genera being increased in relative abundance in samples from NIC patients and five genera being increased in relative abundance from IC patients (Supplementary Figure 12).

Similar to the previous analysis we also wanted to determine the differences between samples from patients that never faced infectious complications and pre and post initial infectious complication samples. We found that the order Burkholderiales was significantly increased in relative abundance in pre-IC samples compared to samples from NIC patients (Supplementary Figure 13). We also found significantly different relative abundances of species between samples from NIC patients and post infectious complication samples. Six of the eight species found to be in increased relative abundance in post-IC patients (Supplementary Figure 13) were the same as the six species found to be in significantly different abundance between samples from NIC and IC patients indicating that post-IC samples may be the driving force for many of these significant associations.

### Multiple Microbial Pathways Are Significantly Different Between NIC Patients and IC Patients

**Figure 4:**
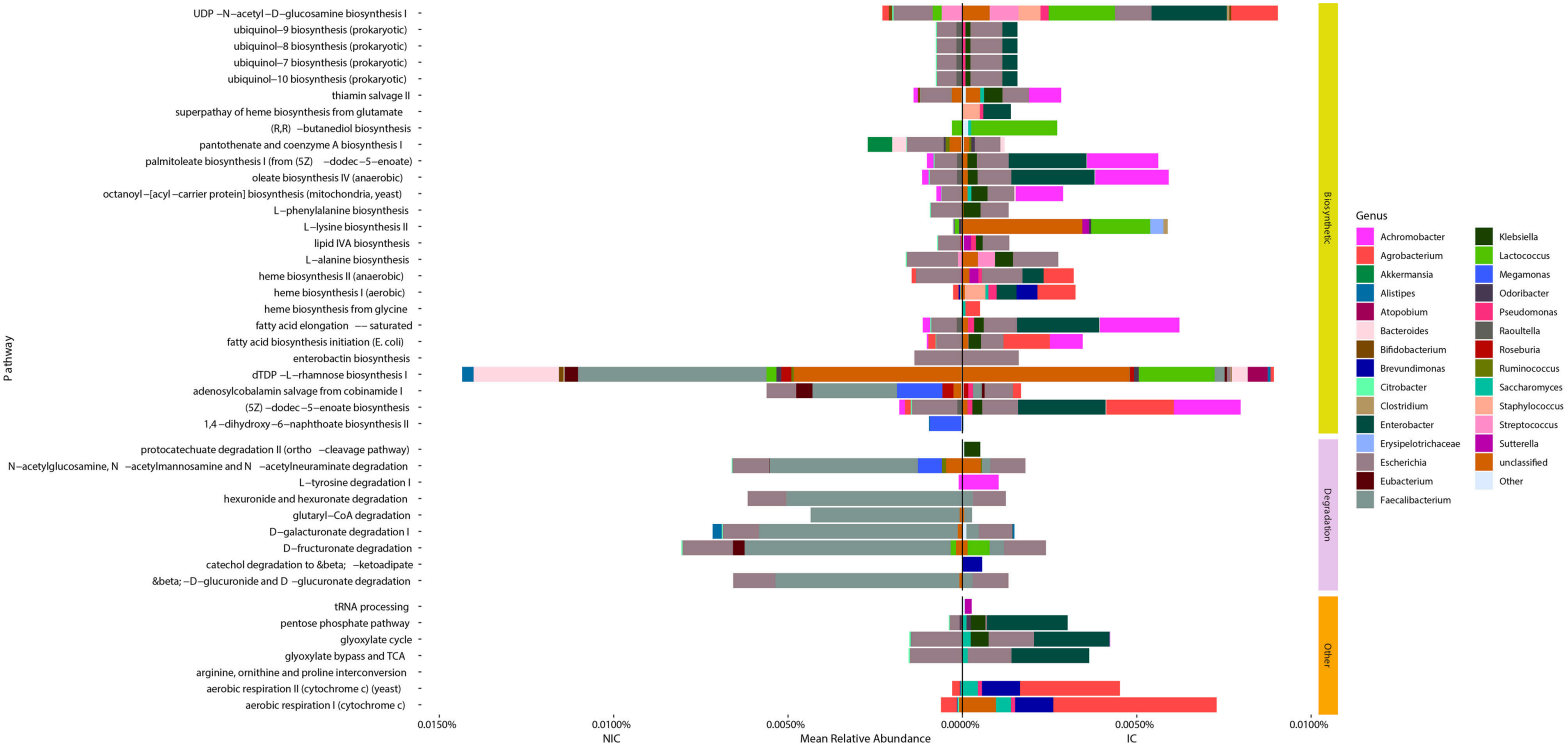
Taxonomic contributions to pathways that were significantly different between samples from NIC patients and IC patients from metagenomic shotgun sequencing. All 42 Metacyc pathways represented in the figure were determined to be significantly different in relative abundance between NIC and IC patients (Wilcoxon rank sum test with correction for false discovery at an alpha value of 0.05). Significantly different pathways were then divided into either being biosynthetic, degradation or other pathway types. Contributions to the relative abundance of these pathways' are colored by genus of the contributing bacteria. Genera that contributed less than a total of 0.01% relative abundance to overall pathways were collapsed into “Other.” Note some pathways have total relative abundances of 0 due to no single species contain all genes required to contribute to the pathway.

We identified 42 microbial MetaCyc pathways that were significantly different in abundance between NIC and IC patients (Figure 4). Interestingly of these 42 pathways, only 11 were increased in NIC patients (Figure 4). Furthermore, of the degradation pathways that were significantly different, 6 out of 9 were in higher relative abundance in samples from NIC patients. This was mainly due to contributions by Faecalibacterium. The majority of biosynthetic pathways were increased in samples from IC patients (Figure 4). Some pathways of interest that were in increased relative abundance in samples from IC patients include aerobic respiration and heme biosynthesis. Of these pathways one interesting note we made was that the mean relative abundance of aerobic respiration I (cytochrome c) was mainly contributed to by bacterial species we found to be significantly different between samples from NIC and IC patients (Figure 4).

Examining the genera that contributed to the differentially abundant functional pathways we found that the reduction of *Faecalibacterium* was a large contributing factor for many of the pathways found in higher abundance in samples from NIC patients (Figure 4). Furthermore, we found that contributions from *Agrobacterium* and *Enterobacter* to multiple different functional pathways in samples from IC patients was a main cause for many pathway abundance level shifts found in NIC patients (Figure 4). Interestingly, we did not find *Enterobacter* as being significantly different in abundance, however this could be due to the low sample size of our cohort.

### Machine Learning Reveals That Bacterial Signatures Are a Strong Classifier for Samples From IC and NIC Patients

**Figure 5:**
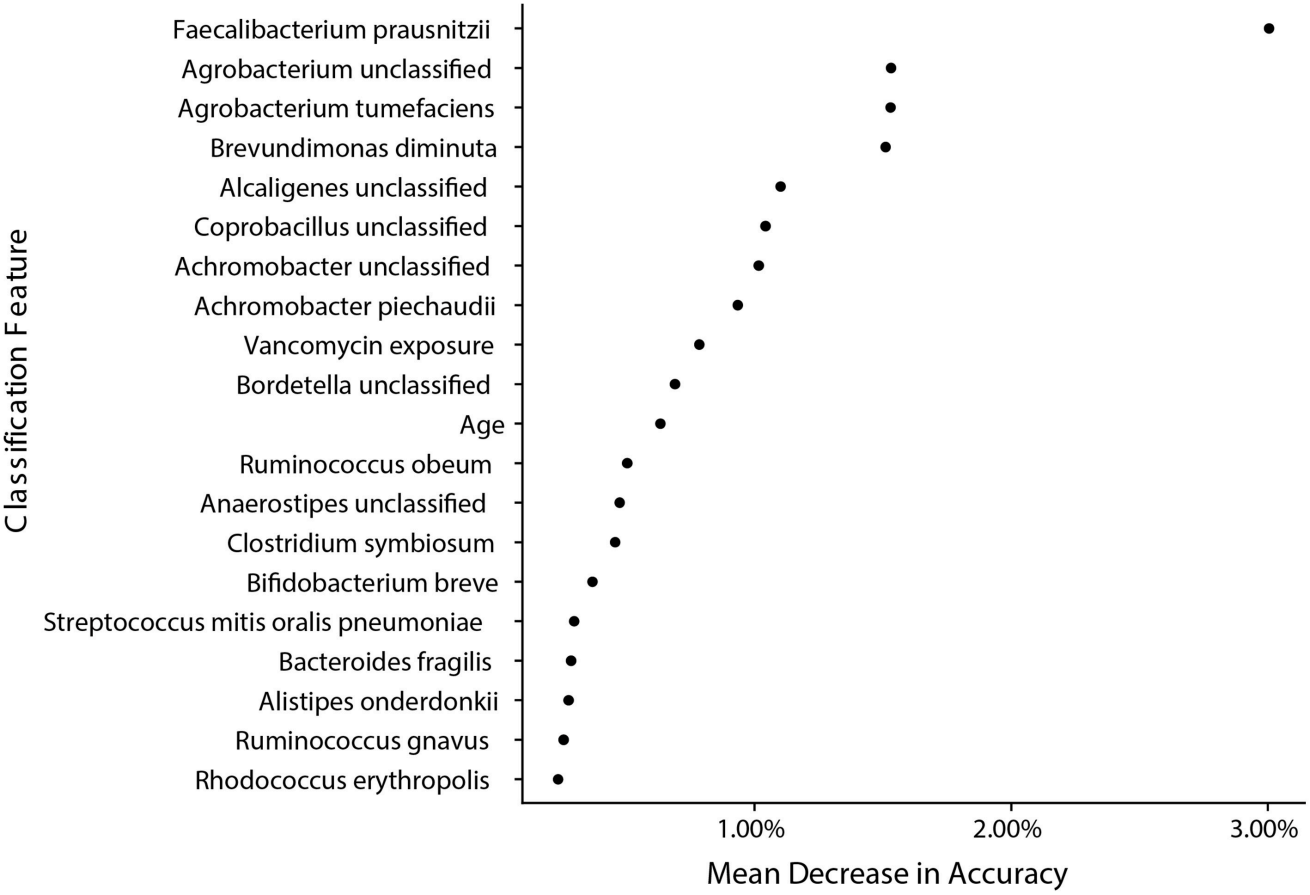
Bacterial species found to be significantly different by metagenomic shotgun sequencing are the most important classification features in a random forest model built from species data and sample metadata. A random forest model with an accuracy of 84.09% built with species information and metadata reveals that species that were found to be significantly different in relative abundance are the most important classification features between samples from NIC and IC patients. The top 20 most important features are ranked by their mean decrease in accuracy when the feature is randomly permuted after model training.

We next wanted to determine how well sample species and metadata could classify samples from IC and NIC patients and determine if the strongest features for classification were similar to taxa already identified to be in significantly different abundance. In this analysis we included all species identified by MetaPhlAn2 and patient metadata (Supplementary File 1). We found the model was able to classify samples as coming from either NIC or IC patients with an accuracy of 84.09% based on the out of bag error rate. Interestingly we found that of the top 20 classification features only 2 were from patient metadata and that the top 5 classification features were all bacterial species that we found to be in significantly different relative abundance between the two patient groups in our previous analysis (Figure 5). Taking a similar approach to try and classify samples from pre-IC patients and NIC patients resulted in accuracies that were not significantly better than random assignment, which may stem from class imbalances and as such more encompassing studies will be required in the future.

To try and better understand if vancomycin exposure was having an impact on the composition of the gut microbiome, we created two different random forest classification models. The first model was trained on and used to classify samples from NIC patients and samples from IC patients that had vancomycin exposure, whereas the other model used samples from NIC patients and IC patients that did not have vancomycin exposure. We found that both models ended up having similar accuracies (76.48 and 80% respectively) and had similar important features (Supplementary Figure 14) indicating a limited effect of vancomycin treatment on gut microbial composition.

### IC Patients Have a Higher Proportion of Gut Microbiota Virulence Factors

**Figure 6:**
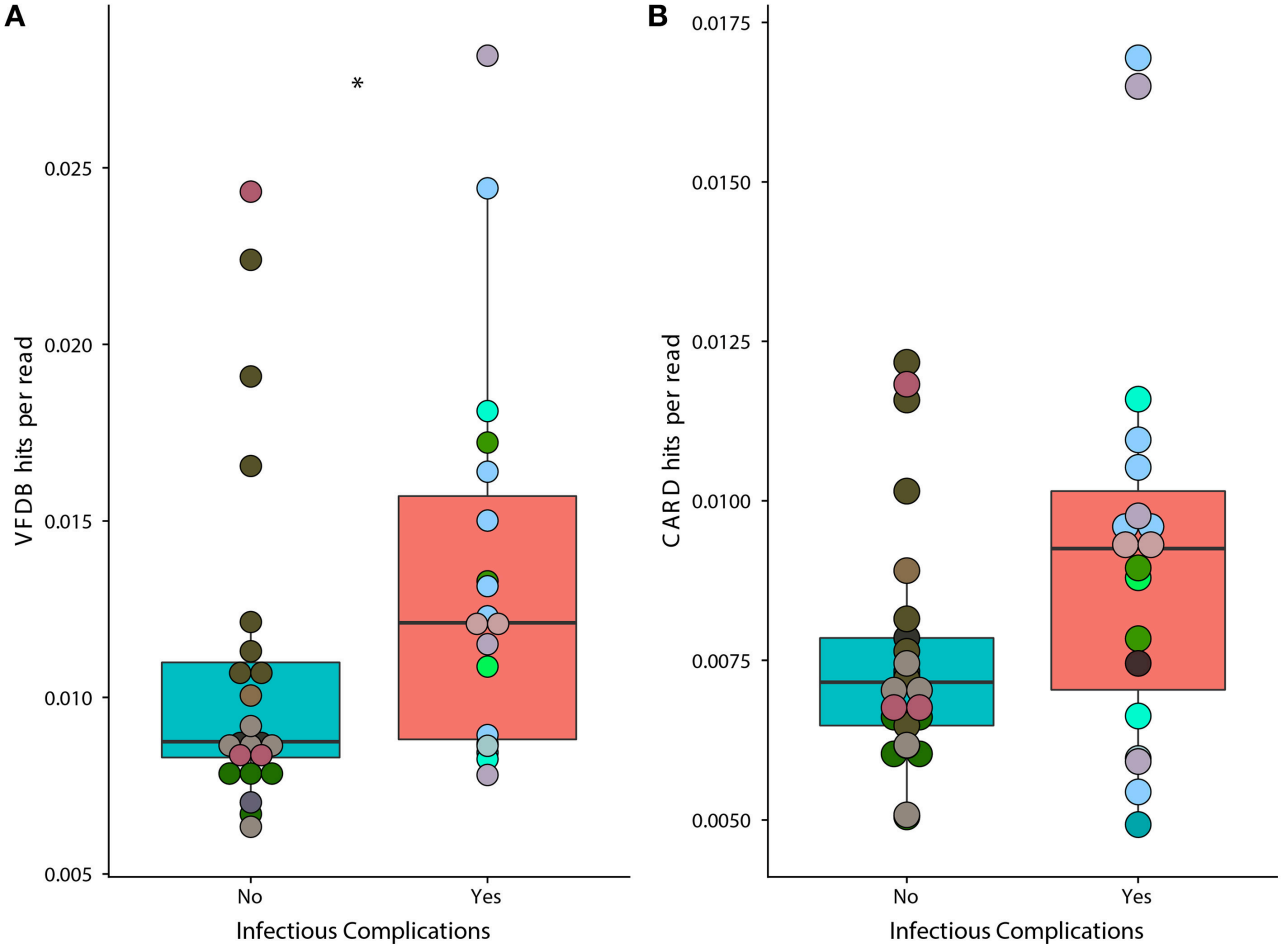
Prevalence of genes associated with virulence factors is significantly higher in samples from IC patients. A higher proportion of metagenomic shotgun sequencing reads were mapped to the Virulence Factor Database **(A)** in IC patients (*p* = 0.02). Proportion of metagenomic shotgun sequencing reads mapped to the Comprehensive Antibiotic Resistance Database **(B)** (*p* = 0.072). Differential prevalence was tested using a Wilcoxon rank sum test with an alpha value of 0.05 (represented by *). Points colored by patient (Supplementary Figure 1).

We found that samples from patients that faced infectious complications had a significant increase in the prevalence of virulence factors according to the Virulence Factor Database (Figure 6A) (Wilcoxon, *p* = 0.02). Overall, we found a total of 134 individual genes that were in differential abundance between samples from IC and NIC patients (Supplementary File 2). The overall prevalence of antimicrobial resistance as described by the Comprehensive Antibiotic Resistance Database was not significantly different between samples from IC and NIC patients (Figure 6B) (Wilcoxon, *p* = 0.072). However, examining the abundance of individual resistance genes did reveal significant differences in samples from IC patients and NIC patients. In total 13 genes found in the CARD database were in differential prevalence between samples form IC and NIC patients (Supplementary File 2). As expected, due to higher levels of antibiotic exposure the vast majority (11 of 13), were increased in samples from IC patients. We did not expect any antimicrobial genes to be more prevalent in NIC patients but found two cepA and mefC, which confer resistance to beta lactams and macrolides, respectively. Examination of the 13 baseline metagenomic samples did not reveal significant differences in the prevalence of anti-microbial resistance genes or virulence factor genes.

## Discussion

The connection between the gut microbiome and infection during chemotherapy treatment has been addressed for multiple different cancer types and cancer treatments (Goodman and Gardner, 2018) including pediatric ALL (Hakim et al., 2018). We describe here the first study to look at associations between pediatric ALL and infection during the first 6 months of therapy using both 16S rRNA gene sequencing data and metagenomic shotgun sequencing data. Similar to previously published work we found significant differences between samples from patients that suffer infectious complications and those that did not, however, in some cases it was unclear whether this was due to antibiotic exposure or the infection itself. We found a significant difference in alpha diversity between the samples from patients that faced infectious complications and those that did not based on phylogenetic diversity. We found multiple taxonomic groups that were significantly higher in relative abundance in samples from IC and NIC patients as well as 42 different metabolic pathways that were significantly higher in relative abundance in either samples from NIC or IC patients. We did not find a significant difference in the total prevalence of antibiotic resistance factors but did find differences in the total prevalence of genes associated with virulence factors within the gut metagenome between NIC and IC patients.

In our study we found significant differences in phylogenetic diversity between samples from IC and NIC patients indicating an association between reduce gut microbial diversity and infectious complications. Furthermore, we found that lower phylogenetic diversity scores were associated with future blood stream infections a result that has been similarly reported in non-Hodgkin's lymphoma (Montassier et al., 2016). These results suggest that monitoring of gut microbial diversity may be useful in determining the future risk of infection for individuals being treated for pediatric acute lymphoblastic leukemia. However, caution is required when interpreting these results due to our small sample size, and other significant confounding variables that we found to be associated with phylogenetic diversity. This includes the age at diagnosis, and the number of days post start of treatment. To control these effects, we were able to show that a logistics regression model based on phylogenetic diversity and other significant confounding variables was able to classify samples from IC or NIC patients with moderate accuracy. As this model is most likely over-fit to our cohort more data is needed from robust studies to build strong classification models for infection. This will allow future models to better account for differences in geographic locations, ethinic backgrounds and diet styles all factors that can affect the microbiome (Yatsunenko et al., 2012). The relationship between alpha diversity and infection was not reported on by Hakim et al. however, they did find that infection was associated with domination by specific families, which could indicate reduce gut microbial diversity.

Examination of the small subset of baseline samples in our cohort revealed that the association between future infectious complications and phylogenetic diversity was near our significant alpha value. Larger numbers of baseline samples will be needed in the future to confirm whether or not gut microbial diversity at diagnosis could serve as a marker for future infectious complications in pediatric ALL. Similar to our whole sample analysis we did find subsequent bloodstream infections to be significantly associated, indicating that gut microbiome diversity at diagnosis may be useful in determining the future risk of blood stream infections during chemotherapy treatment.

One difficulty in studying the relationship between the composition of the gut microbiome and pediatric ALL is the usage of antibiotic prophylaxis due to immune suppression. All patients receive Septra, a broad-spectrum prophylactic antibiotic, throughout treatment as prophylaxis for pneumocystis opportunistic infections and the vast majority of patients will receive various other antibiotics at the first sign of possible infection (i.e., a fever). We expected this to have a large impact on gut microbial alpha diversity, however, the only antibiotic we found to be significantly associated with phylogenetic diversity was vancomycin. The majority of patients that received vancomycin were also patients with infectious complications making it difficult to determine how much of an impact infection vs. vancomycin had on the phylogenetic diversity of the samples; especially since vancomycin treatment is usually reserved for severe infections, which could be having dramatic effects on the gut microbiota. Furthermore, all patients that received vancomycin received it intravenously, which has limited gut availability in mice models (van Oosten et al., 2013). However, it should be noted that as described previously many of these individuals will have reduced structural integrity of the gut barrier, which may allow the passage of vancomycin into the gut. While only one statistically significant association between phylogenetic diversity and specific antibiotics was observed, the usage of drugs other than piperacillin-tazobactam were significantly associated with infectious complications and due to the small sample size may still play a role in the differences observed between NIC and IC patients.

Further analysis also revealed significant differences in beta diversity between individuals that received vancomycin and individuals that received anti-fungal medication (Supplementary Figure 9). Due to the limited number of samples exposed to anti-fungal medication (*n* = 5) and three of the five samples coming from the same patient it is difficult to determine whether anti-fungal exposure or interpatient variability between samples is the driving factor of significance for this result. Vancomycin exposure on the other hand was found in samples from multiple patients. To determine whether vancomycin exposure was a better classifier than gut microbiota species composition we ran random forest models containing both species abundance and metadata characteristics. We found that vancomycin exposure was in the top 10 classification features but was below all of the species that we found to be significantly different between samples from IC and NIC patients. Further analysis on the separate classification models for IC patients that received vancomycin or patients that did not receive vancomycin also revealed similar important features (Supplementary Figure 14). This indicated that underlying differences in the gut microbiome between IC and NIC patients are present despite vancomycin usage, which could be due to the infection itself or the combination of other anti-biotic treatments.

Examining further differences in beta diversity we found a significant separation between samples from NIC and IC patients. We found in multivariate testing, that infectious complications within the first 6 months of therapy was the largest explanatory variable (Table 3), although it was not the only significant explanatory variable, in univariate analysis (Table 2). Our second biggest explanatory variable was sex; however, we believe this is most likely due to all females in our study experiencing an infectious complication, and therefore it does not actually represent a difference due to sex. This finding will need to be looked at in the future as differences in gut microbiome composition due to sex is not completely understood (Haro et al., 2016). We did not find any significant difference in phylogenetic diversity between male and female samples at baseline or collectively. Finally, looking at samples based on pre and post initial infectious complications we found that both pre and post IC samples were significantly different then NIC samples indicating that beta diversity may be useful in determine risk for infection in the future. Samples from patients 15 and 8 both grouped with NIC samples before infection and had follow-up samples that were post infection and grouped with other IC samples indicating a dramatic shift in their gut microbial community (Figure 2B). This could have been due to multiple factors including anti-infectious treatments or the infectious complication itself. Interestingly, three patients (4, 12, and 13) that were pre-infection, grouped with samples that already had an infection (Figure 2B). Examination of the metadata from these patients did not reveal any key differences other than patient 13 received both vancomycin and caspofungin before infection, which could be the cause of their similar gut microbial community to other individuals with infections. Pre-IC samples from patients 4, 8, 12, and 15 had all only been exposed to piperacillin-tazobactam and so differences in antibiotic treatment did not explain why two individuals grouped with NIC samples and two grouped with IC samples.

Examining the abundance of different taxonomic phyla's revealed that NIC patients had increased relative abundances of Bacteriodetes whereas IC patients had increased levels of Proteobacteria. This increase in Proteobacteria was similar to findings by Hakim et al., who saw that at baseline increased levels of Proteobacteria was predictive of subsequent febrile neutropenia. Evaluating their suggested model of a relative abundance over 0.01% being predictive of febrile neutropenia, was not effective in our dataset as all samples contained Proteobacteria relative abundances above this cut-off. We did however see that a cut-off value of 1% relative abundance in the baseline 16S rRNA gene sequencing dataset did result in all patients below the cut-off not having future infections and 5/7 patients above the cut-off acquiring future infections (Fisher's Exact test; *p* = 0.06993). These findings do suggest that Proteobacteria may play a role in the prediction of future infections but the relative abundance cut-off for using this phylum may vary cohort to cohort. This could be due to multiple differences such as the variable region sequenced or the geographic location of treatment. Further evidence of Proteobacteria playing an important role in future infectious complications was seen in the significantly differential abundance of Burkholderiales (an order of bacteria that belong to the Proteobacteria phylum) in pre-IC samples compared to NIC samples.

Further comparisons of Hakim et al.'s findings of the predictive nature of the domination (relative abundance < 30%) of Enterococcaceae or Streptococcaceae revealed varied results. No samples within the cohort were dominated by Enterococcaceae and so this predictive marker was of no use in our cohort, however, of the 7 samples dominated by Streptococcaceae five were from IC patients (*p* = 0.2117). We did not find any significant relationship between Streptococcaceae domination and subsequent gastrointestinal infections (*p* = 0.233), blood stream infections (*p* = 0.1563), or febrile neutropenia (*p* = 0.1827). However due to the small sample size of our cohort it could be due to low statistical power.

We found multiple species that were significantly different between samples from NIC and IC patients five of six which, were increased in relative abundance in samples from IC patients. Furthermore, many of these bacterial species have been described as opportunistic pathogens in immunocompromised individuals. *Brevundimonas diminuta*, has previously been described as an opportunistic pathogen found in patients undergoing chemotherapy treatment (Han and Andrade, 2005). Bacteria in both *Achromobacter* and *Alcaligenes* genera have also been described as uncommon opportunistic pathogens in patients with various cancers including hematological malignancies (Aisenberg et al., 2004). Possibly suggesting the gut microbiome as a source of infection from these bacteria. It should be noted that some of these species were only found in a subset of patients and so the presence of these bacteria was not ubiquitous across all IC patients. Future studies will need to be done to determine how common these bacteria are in patients with infectious complications and which role they are playing in the gut including and not limited to their colonization potential. As many of these species are normally found on and within plants it's possible that they represent bacteria found within the diet.

The only species increased in relative abundance in samples from NIC patients was *Faecalibacterium prausnitzii* a bacterial species that has been shown to be decreased in relative abundance in type II diabetes, ulcerative colitis, and Crohn's disease (Lopez-Siles et al., 2017). *F. prausnitzii* is an abundant member of the gut microbiome and has previously been shown to be found in the gut microbiome of pediatric ALL patients (Rajagopala et al., 2016). *F. prausnitzii also* has both anti-inflammatory properties (Lopez-Siles et al., 2017) and produces butyrate and propionate, short chain fatty acids that have been shown to be important in host metabolism and immune function (den Besten et al., 2013; Corrêa-Oliveira et al., 2016). The reason as to why this prominent member is reduced in samples from IC patients remains to be solved but could be due to its high sensitivity to oxygen, which could be higher than normal in samples from IC patients as indicated by the increase in relative abundance of aerobic respiration in the gut metagenome (Figure 4). All of the species that were significantly increased in relative abundance in IC patients were aerobic bacteria, which all contributed to the majority of the abundance of aerobic respiration pathways in samples from IC patients (Figure 4). One possible cause of increased oxygen availability is the higher than normal reduction of gut barrier integrity and low-grade intestinal blood loss, in IC patients from chemotherapeutics (Blijlevens et al., 2000) or the infectious complication, allowing oxygen into the gut.

We found increased prevalence of genes annotated as virulence factors, such as iron acquisition, and specific antimicrobial resistance genes in both samples from NIC and IC patients. However, we did not find a difference in the prevalence of these genes at baseline indicating that this difference may be due to differences in treatment between NIC and IC individuals. These differences could be due to anti-infectious treatment or prolonged hospital stays for IC patients, which may increase the likely hood for the colonization of pathogenic bacteria within the gut microbiome. Interestingly, in samples from NIC patients we found increased prevalence of two genes cepA and mefC, which confer resistance to beta lactams and macrolides, respectively (Jia et al., 2017). We did not expect to find any genes significantly increased in prevalence in samples from NIC patients. One explanation for the relative increase in cepA prevalence could be due to it being found within members of the *Bacteroides* genera (Rogers et al., 1994) a group of organisms found to be enriched at the genera level (Supplementary Figure 12) within samples from NIC patients. We found multiple antimicrobial genes that were significantly, increased in prevalence in samples from IC patients that conferred resistance to a large array of antibiotics. We believe this is due to the increased exposure of IC patients to antibiotics driving antimicrobial resistance.

We have reported multiple significant associations between the gut microbiome and infectious complications in pediatric ALL patients, however, in some cases it was not possible to determine whether infection itself, anti-biotic treatment, or a combination of the two were the driving factors for these associations. We were able to create a model with high classification accuracy of infectious complications, based on phylogenetic diversity, while acknowledging that the complexity of pediatric ALL treatment may be confounding some of these associations. We found distinct bacterial groups that differ between patients that faced infection and those that did not face infection, furthermore, we found that patients that face infection have higher proportions of genes annotated as virulence factors within their gut metagenome. This may indicate that perhaps harboring these communities within the gut increases the patient's susceptibility to infection during therapy or that infection itself increases the risk of carrying higher prevalence of virulence factors within the gut metagenome. Further studies will be needed to determine the connection between the gut microbiome and risks for infection during chemotherapy, to create robust models for the stratification of the risk of infection for patients undergoing treatment for pediatric ALL.

## Data Availability

The datasets generated for this study can be found in the European Nucleotide Archives under the accession number PRJEB29237. Custom R scripts used to analysis amplicon sequence variant. HUMAnM2, and MetaPhlAn2 data can be found at https://github.com/nearinj/ALL_Infection_and_the_Microbiome. Sample metadata can be found in Supplementary File 1.

## Ethics Statement

All patients/guardians provided informed written consent to participate in this study. The protocol for this study was approved by the IWK Health Centre Research Ethics Board (REB #1019670).

## Author Contributions

JV, TM, KK, and ML designed the study and obtained funding. JC, TM, and KK recruited patients, conducted chart reviews, and collected samples. KK also participated in data analysis and is the principal investigator for the study. SW cataloged, stored, and extracted DNA for all samples. JN carried out computational experiments and conducted all bioinformatics and data analysis including generation of figures and writing of the manuscript. All authors read and approved the final manuscript.

### Conflict of Interest Statement

The authors declare that the research was conducted in the absence of any commercial or financial relationships that could be construed as a potential conflict of interest.
