# Supplementary material for: Infectious Complications Are Associated With Alterations in the Gut Microbiome in Pediatric Patients With Acute Lymphoblastic Leukemia
Source: Front Cell Infect Microbiol. 2019 Feb 19;9:28. doi: 10.3389/fcimb.2019.00028 (PMC6389711; doi:10.3389/fcimb.2019.00028)
Supplement: Supplementary file 2 [file Data_Sheet_2.pdf]

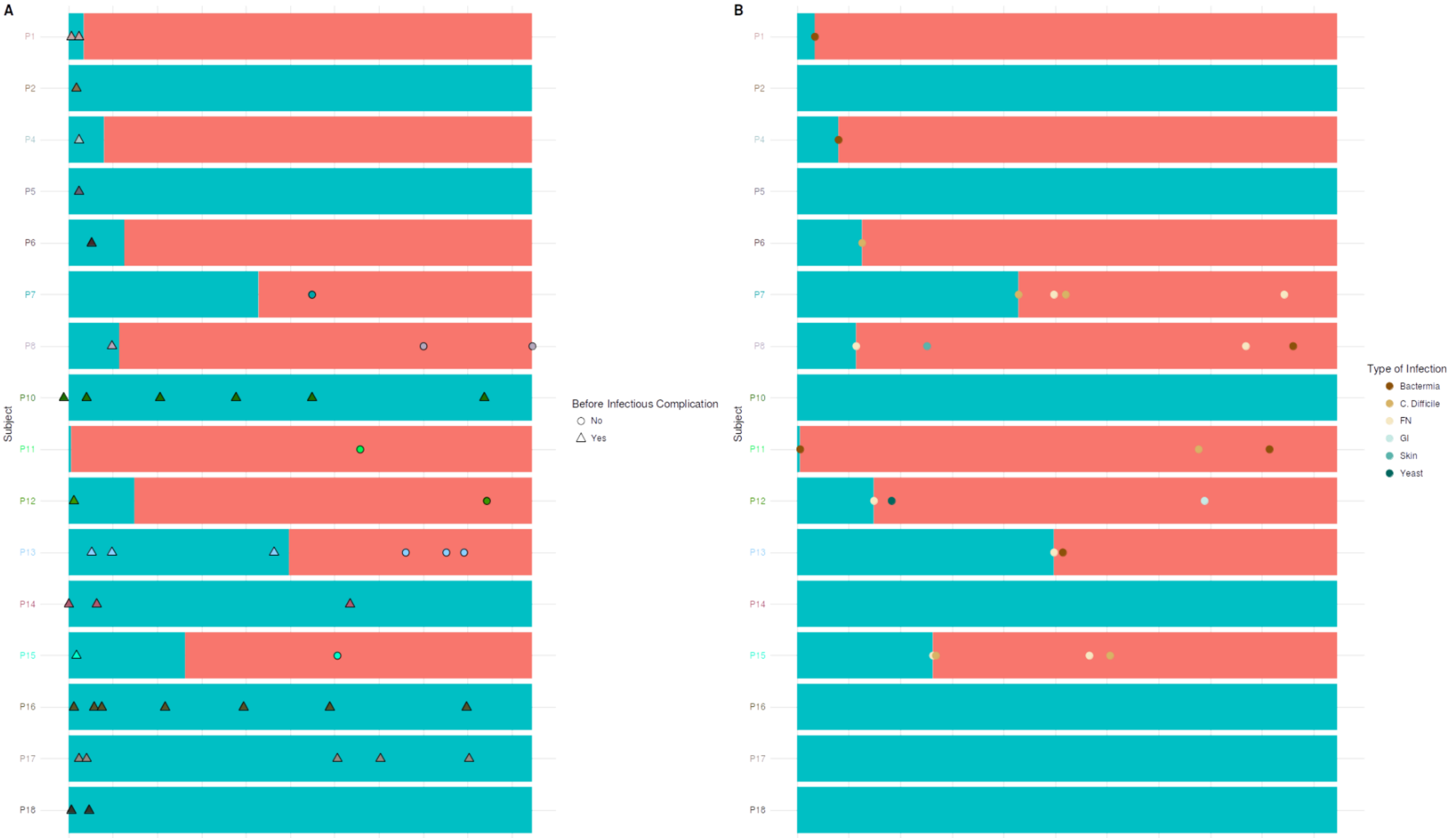

Supplemental Figure 1: **Patient sampling times and initial infectious complication events over the first six months of therapy**.

A swimmer chart representing the first six months of treatment for patients within the study. Patients were numbered based on study ID with some ID's being skipped due to failure to collect samples. A) Points represent when a sample was collected, and color change within the bars represent an initial infectious complication. Points and labels on the plot are colored by patient and are consistent across all figures in the manuscript. B) Points represent the occurrence of an infectious complication colored by the type of infectious complication. Bars represent the timing of an initial infectious complication.

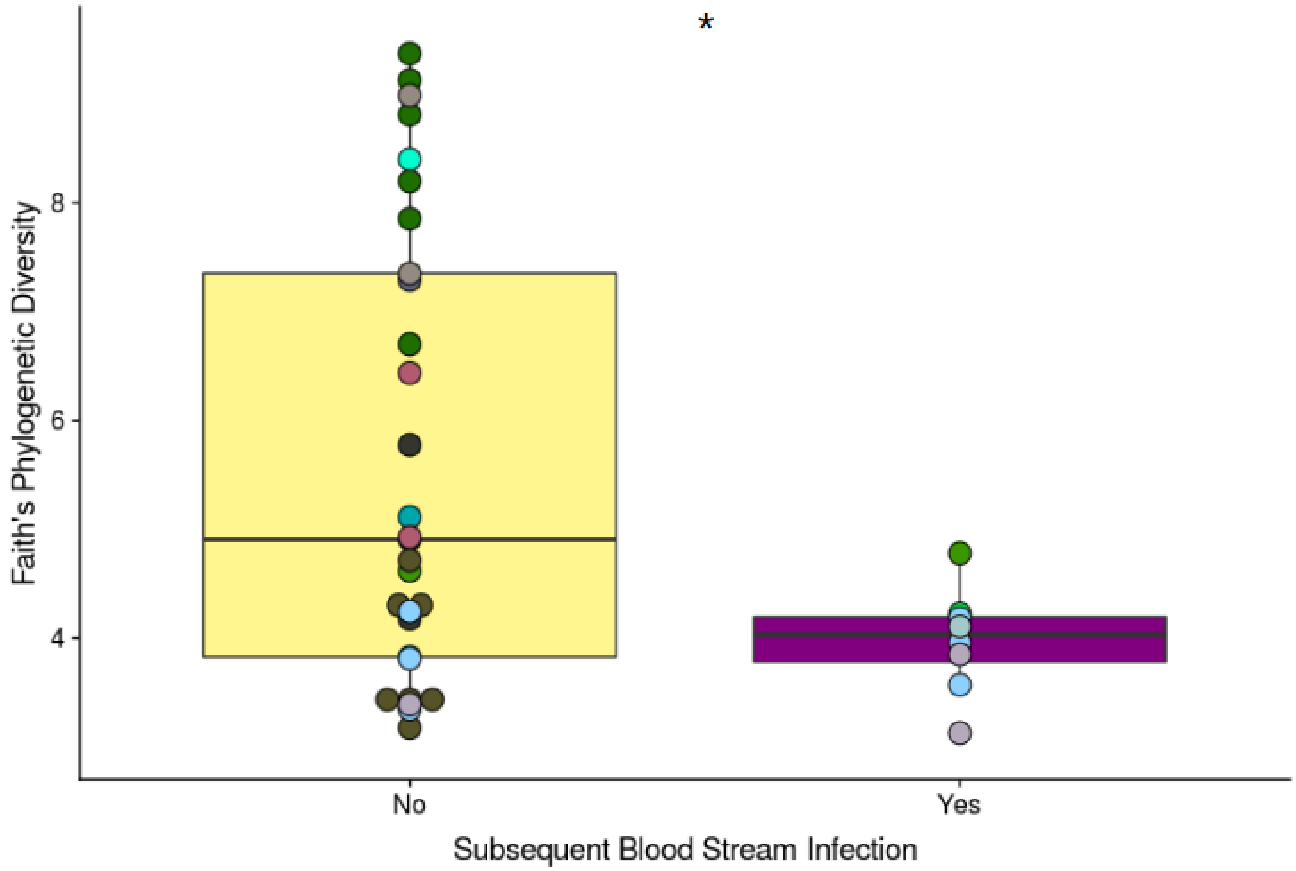

Supplemental Figure 2: **Lower phylogenetic diversity is associated with subsequent bloodstream infections.**

Samples were divided into those from patients that had subsequent blood stream infections and those that did not. A total of eight samples (from five different patients) were taken before a patient encountered a blood stream infection in the future and 29 samples were taken from patients that didn't face subsequent bloodstream infections (**Sup File 1**). Significances was tested with a Wilcoxon rank sum test at an alpha value of 0.05. Dots are colored by the patient that the sample was collected from (**Sup Fig 1**). This data was generated from 16S rRNA gene sequencing data.

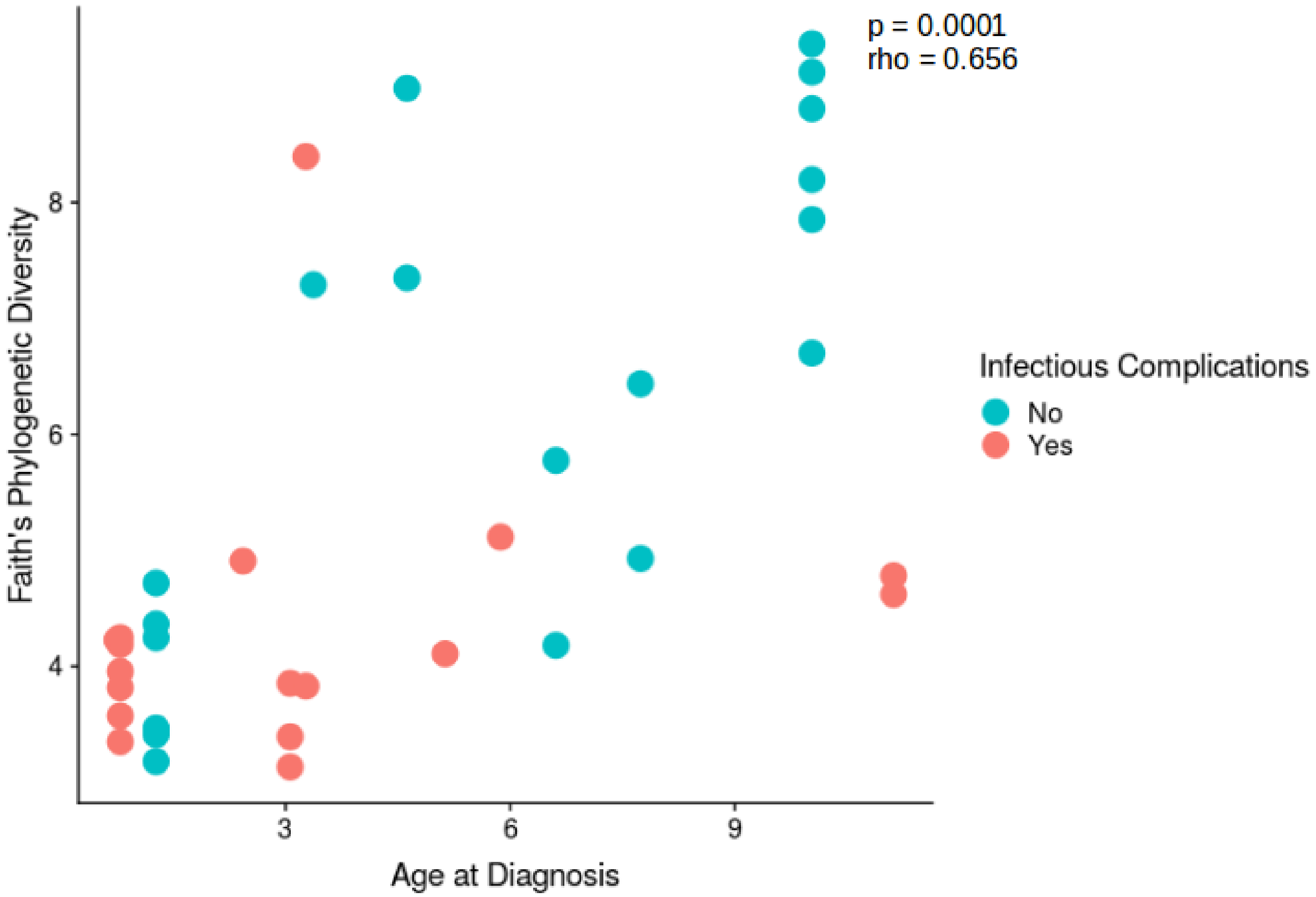

Supplemental Figure 3: **Age at diagnosis is significantly associated with phylogenetic diversity.** A significant spearman correlation was found between the age of the patient the sample was collected from and their phylogenetic diversity score. Samples were colored based on whether they came from an NIC or IC patient. This data was generated from 16S rRNA gene sequencing data.

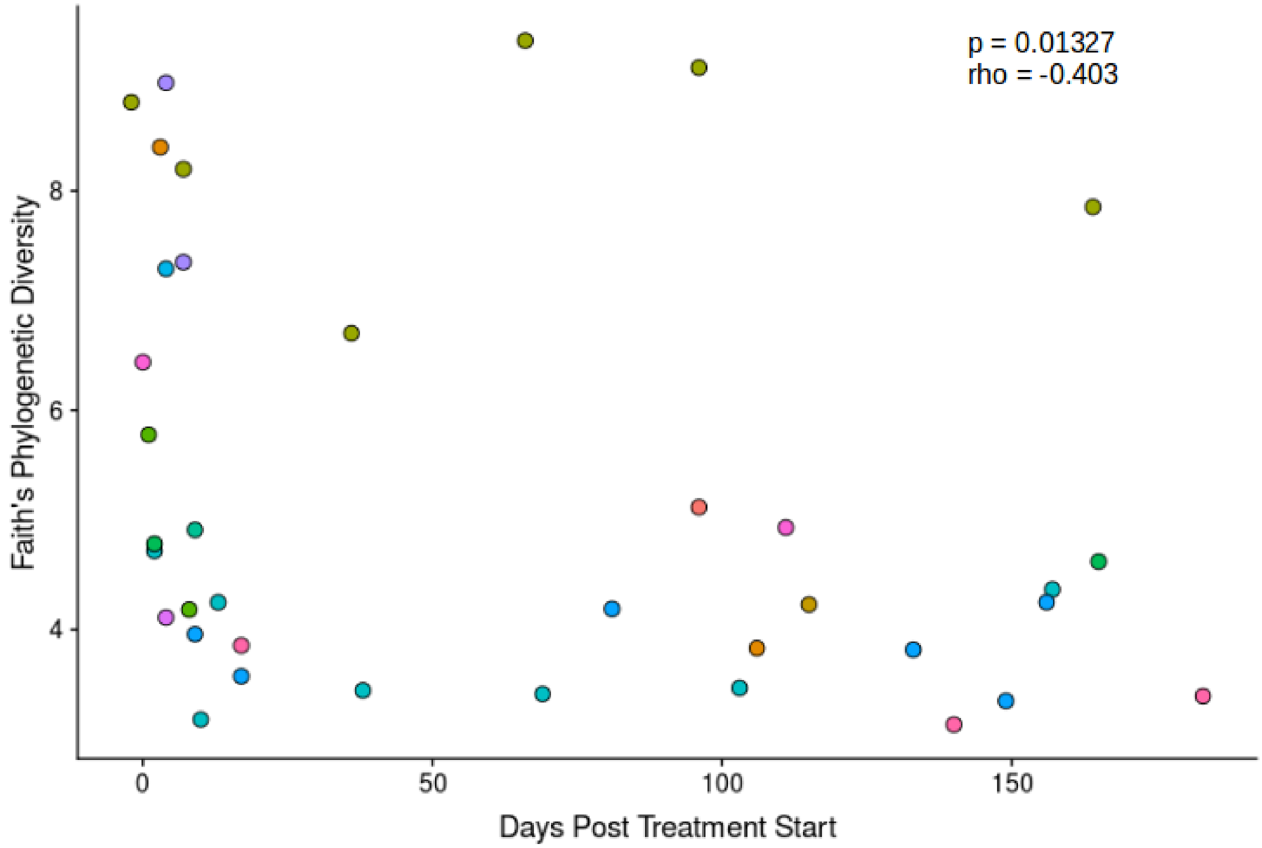

Supplemental Figure 4: **Days post treatment start is significantly associated with phylogenetic diversity.**
A significant negative spearman correlation was found between days post treatment start and phylogenetic diversity indicating an effect of treatment of gut microbial diversity. Samples are colored based on the patient that they were collected from (**Sup Fig 1**). This data was generated from 16S rRNA gene sequencing data.

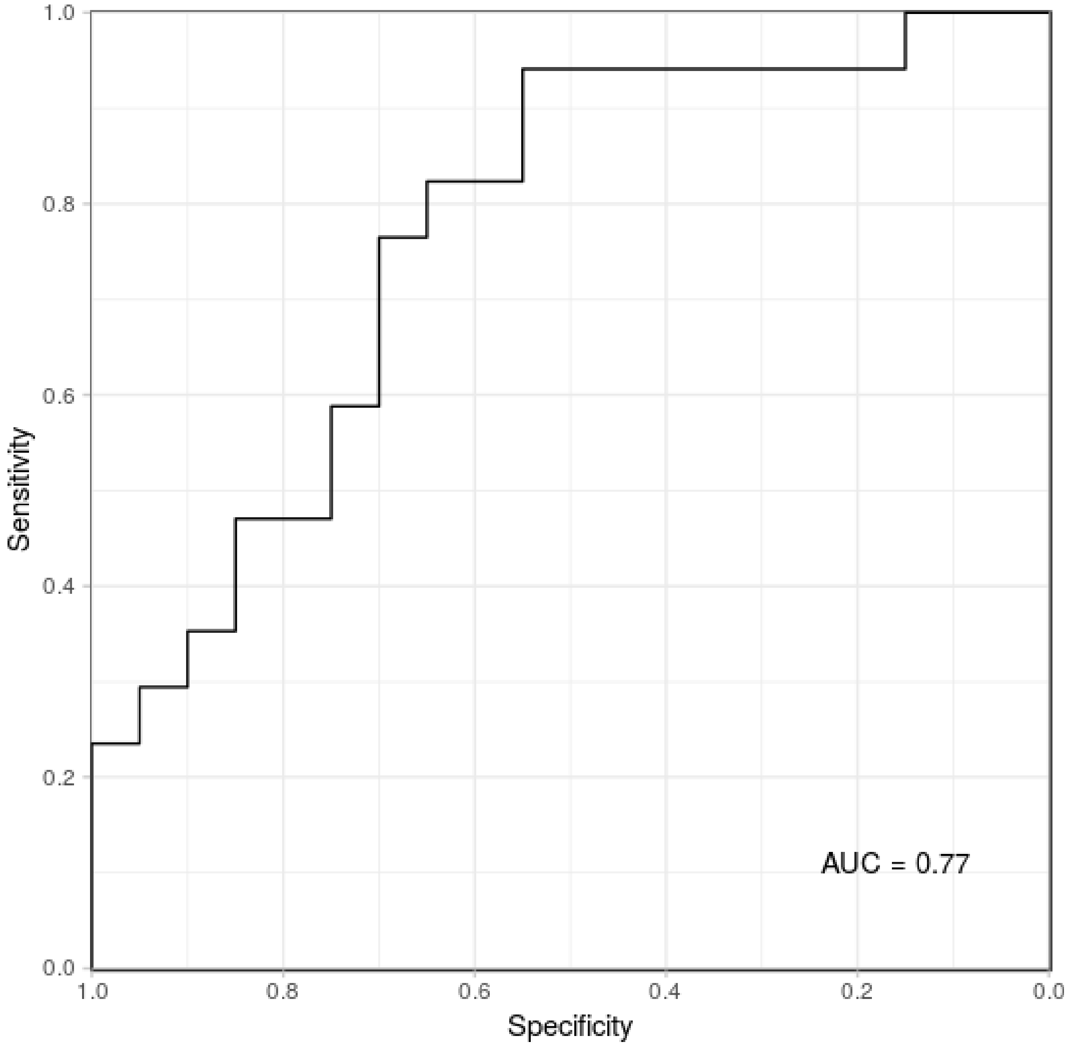

Supplemental Figure 5: **Classification of samples from either NIC or IC patients based on Faith's phylogenetic diversity and adjusted for collection time, age at diagnosis and treatment type.**
A receiver operating characteristic (ROC) curve representing the classification accuracy of samples from either being collected from NIC or IC patients using a logistic regression model based on Faith's phylogenetic diversity and adjusted for collection time and treatment type. Overall an area under the curve of 0.77 was obtained through this model and Faith's phylogenetic diversity was found to be close to our significant alpha value p=0.0994.

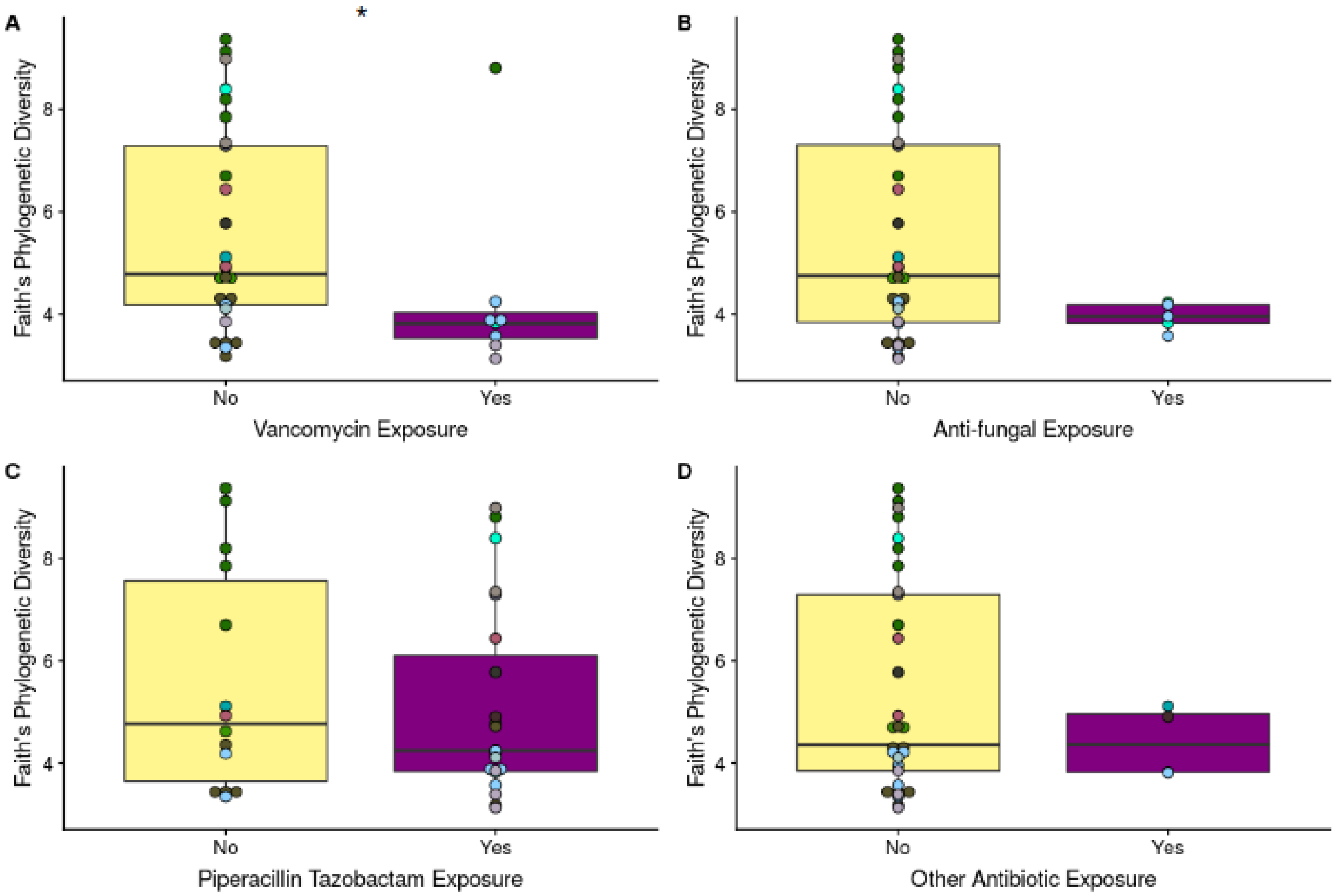

Supplemental Figure 6: **Phylogenetic diversity is significantly associated with vancomycin exposure.**

Differences in phylogenetic diversity was compared for samples exposed to various antibiotics including vancomycin A), anti-fungal medication B), piperacillin tazobactam C), and other less commonly used antibiotics D). Significance was determined using a Wilcoxon rank sum test with an alpha value of 0.05. Note that due to sparse use of some medications such as anti-fungal medication it is difficult to tell their impact on phylogenetic diversity. This data was generated from 16S rRNA gene sequencing data.

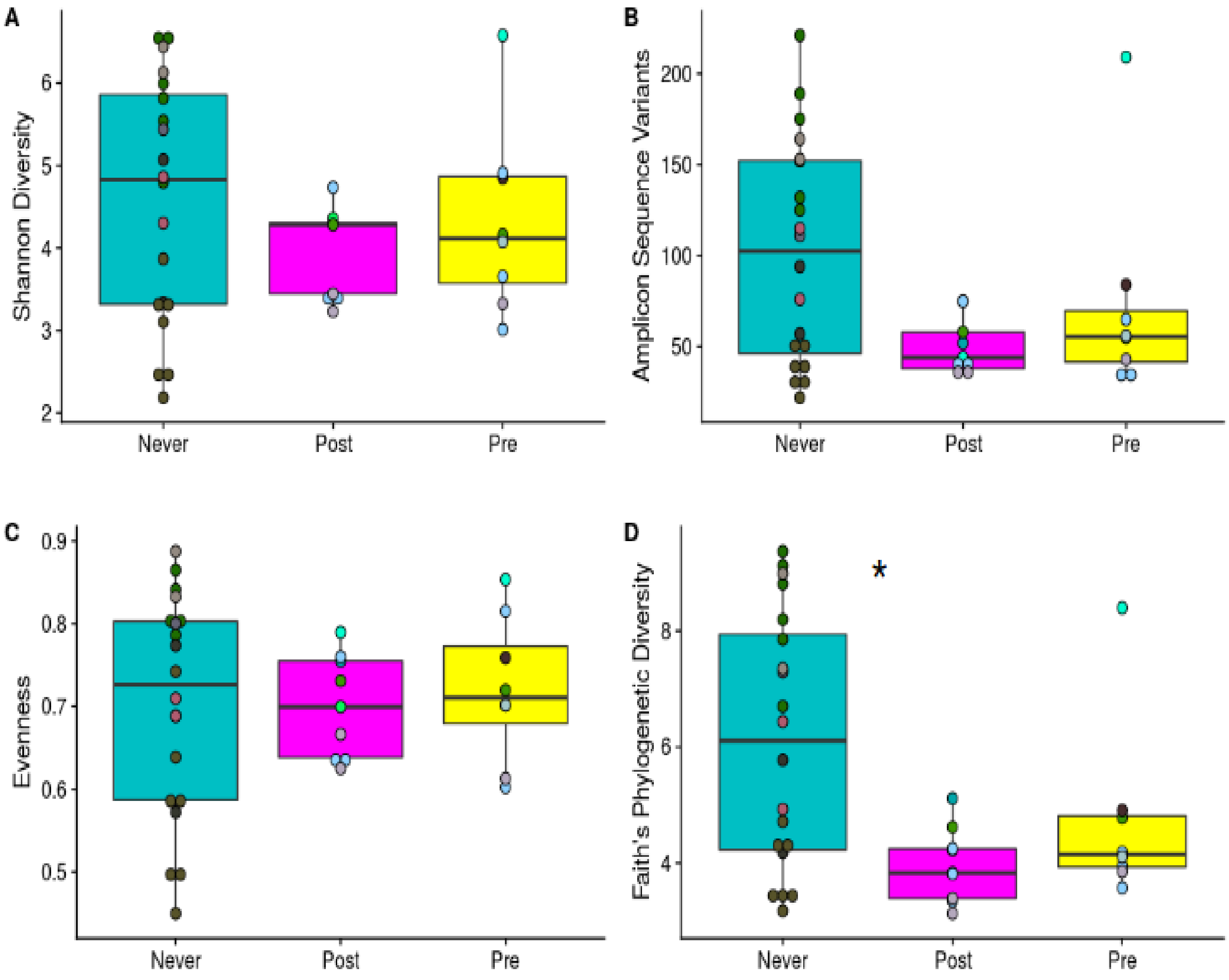

Supplemental Figure 7: **Phylogenetic Diversity is significantly different between samples from NIC patients and post infectious complication samples.**
Differences in alpha diversity between samples from NIC patients and pre and post infectious complication samples from IC patients (pairwise Wilcoxon rank sum tests at an alpha value of 0.05). A significant difference in phylogenetic diversity was observed between samples from NIC patients and post infectious complication samples from IC patients (represented by *). Points are colored by individual (Sup Fig 1). This data was generated from 16S rRNA gene sequencing data.

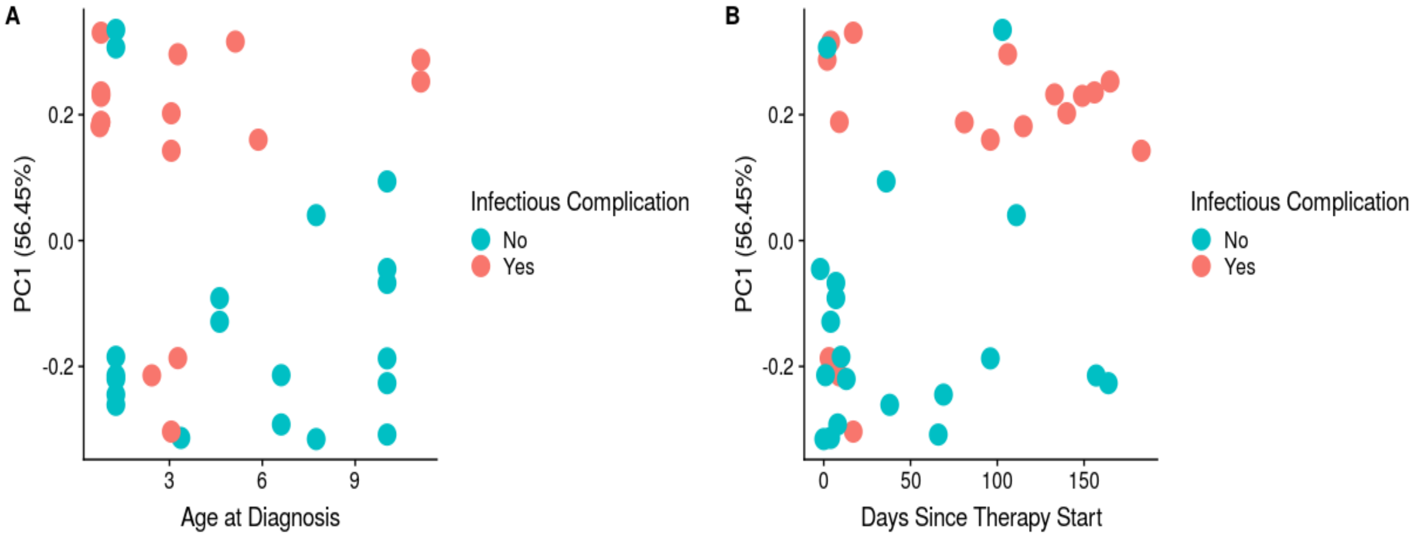

Supplemental Figure 8: **Weighted UniFrac beta diversity is significantly associated to days since therapy start but not age at diagnosis.**

Examining the relationship between beta diversity, days since therapy start and age at diagnosis revealed that despite both age and time since therapy start being significantly related to alpha diversity only days since therapy start was significantly related to weighted UniFrac beta diversity (PERMANOVA: $p = 0.032$, $r^2 = 0.089$) (Table 2). This data was generated from 16S rRNA gene sequencing data.

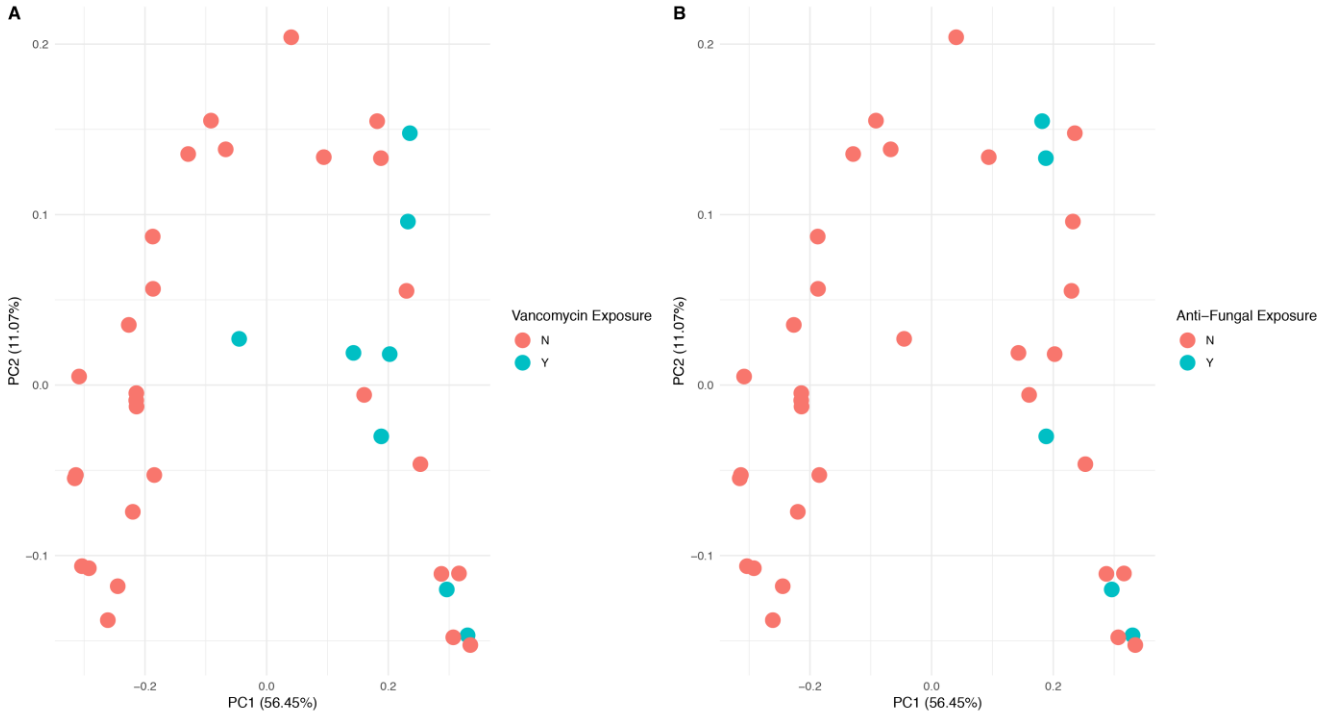

Supplemental Figure 9: **Weighted UniFrac beta diversity colored by exposure to vancomycin or anti-fungal medication.**

Principal coordinates of analysis plots of the weighted UniFrac distances between samples. Samples are colored by Vancomycin exposure within 2 weeks of collecting the samples A) or anti-fungal medication exposure within 2 weeks of collecting the samples B). This data was generated from 16S rRNA gene sequencing data.

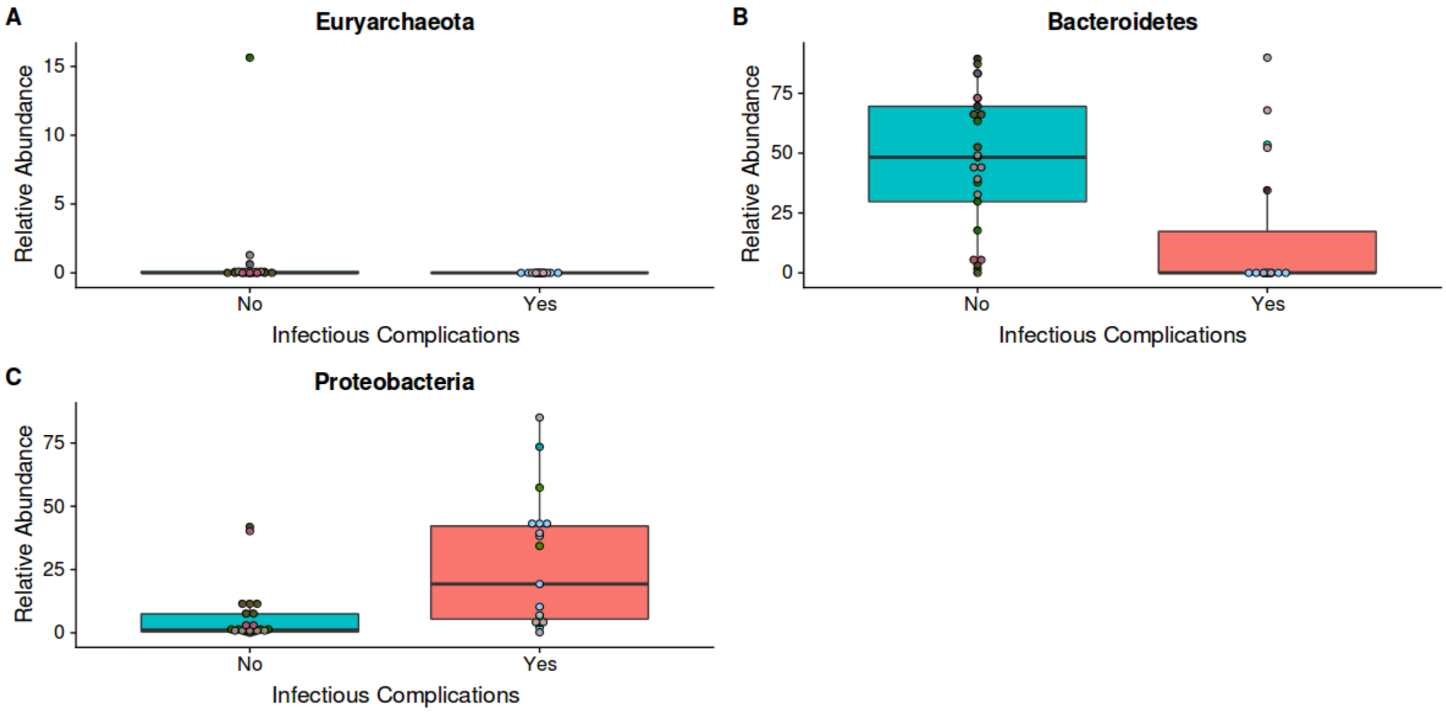

Supplemental Figure 10: **Three phyla are significantly different in abundance between samples from NIC and IC patients.**

Both Euryarchaeota and Bacteroidetes were found to be significantly increased in relative abundance among samples from NIC patients. The phylum Proteobacteria was found to be increased in samples from patients that faced infection. (Wilcoxon rank sum test with correction for false discovery at an alpha value of 0.05). Points are colored by patient (**Sup Fig 1**). This data was generated from metagenomic shotgun sequencing data.

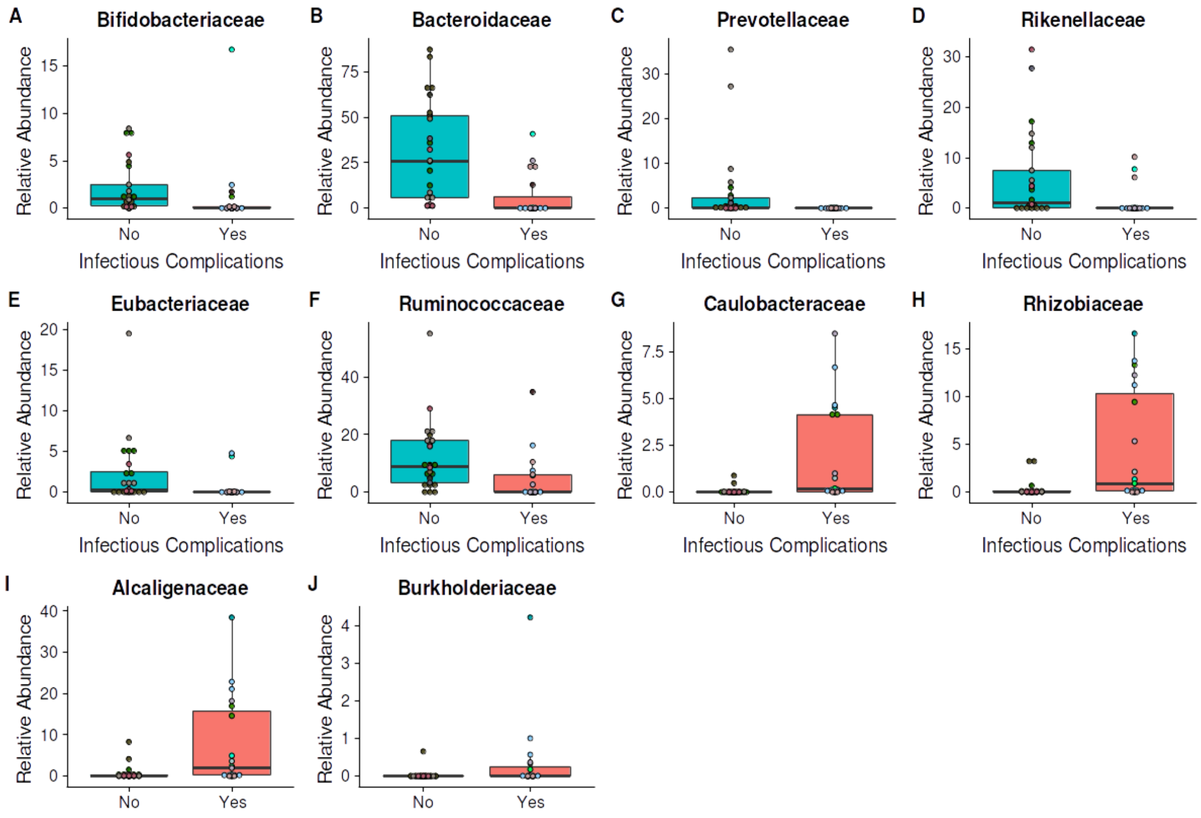

Supplemental Figure 11: **Multiple Families are significantly different in abundance between samples from NIC and IC patients.**

Six families were found to be in significantly increased relative abundance in samples from NIC patients. Four families were found to be in significantly increased relative abundance in samples from IC patients. (Wilcoxon rank sum test with correction for false discovery at an alpha value of 0.05). Points are colored by patient (**Sup Fig 1**). This data was generated from metagenomic shotgun sequencing data.

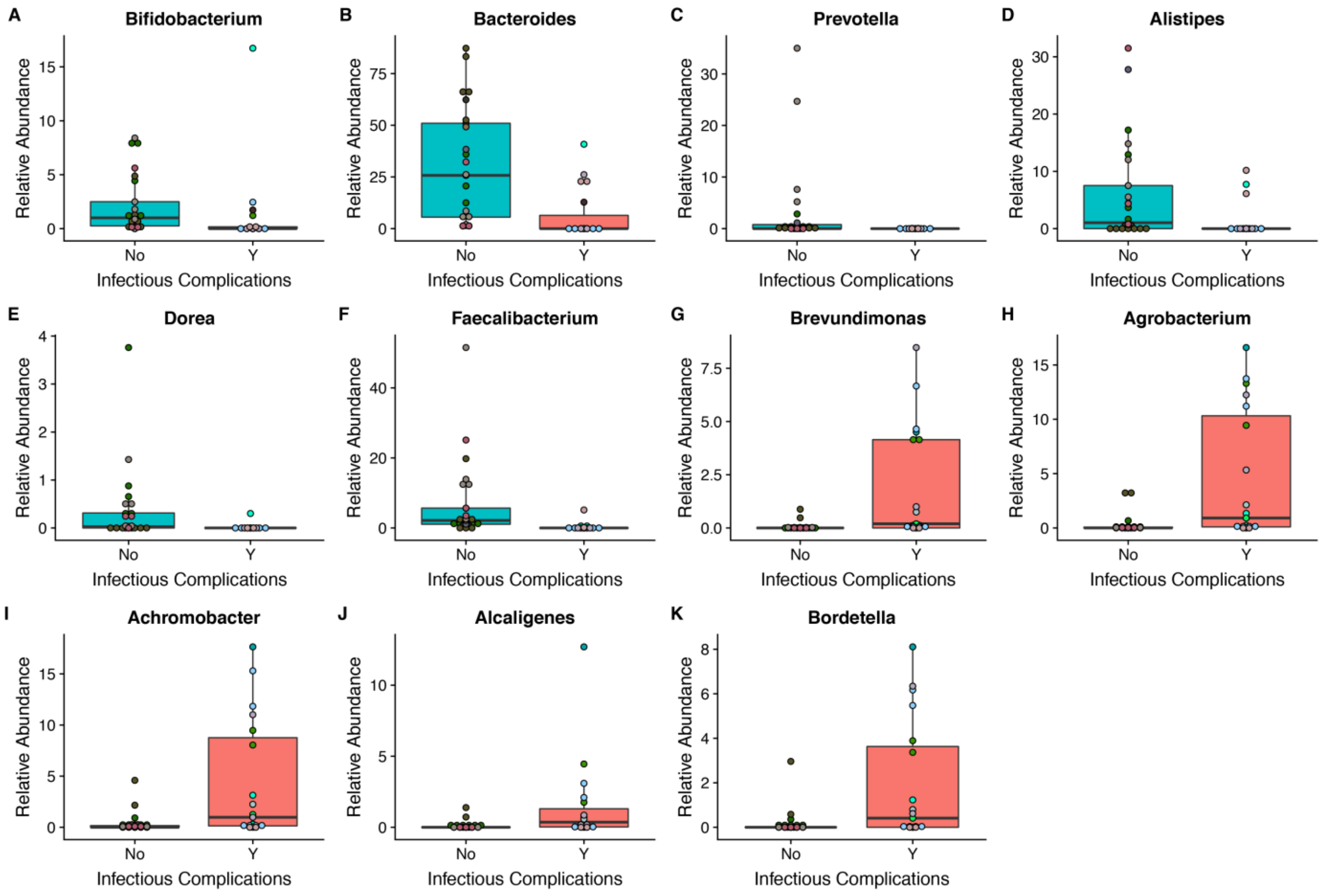

Supplemental Figure 12: **Multiple genera are significantly different in abundance between samples from NIC and IC patients.**

Eleven genera were found to be significantly different in relative abundance between samples from IC and NIC patients. (Wilcoxon rank sum test with correction for false discovery at an alpha value of 0.05). Points are colored by individual patient (**Sup Fig 1**). This data was generated from metagenomic shotgun sequencing data.

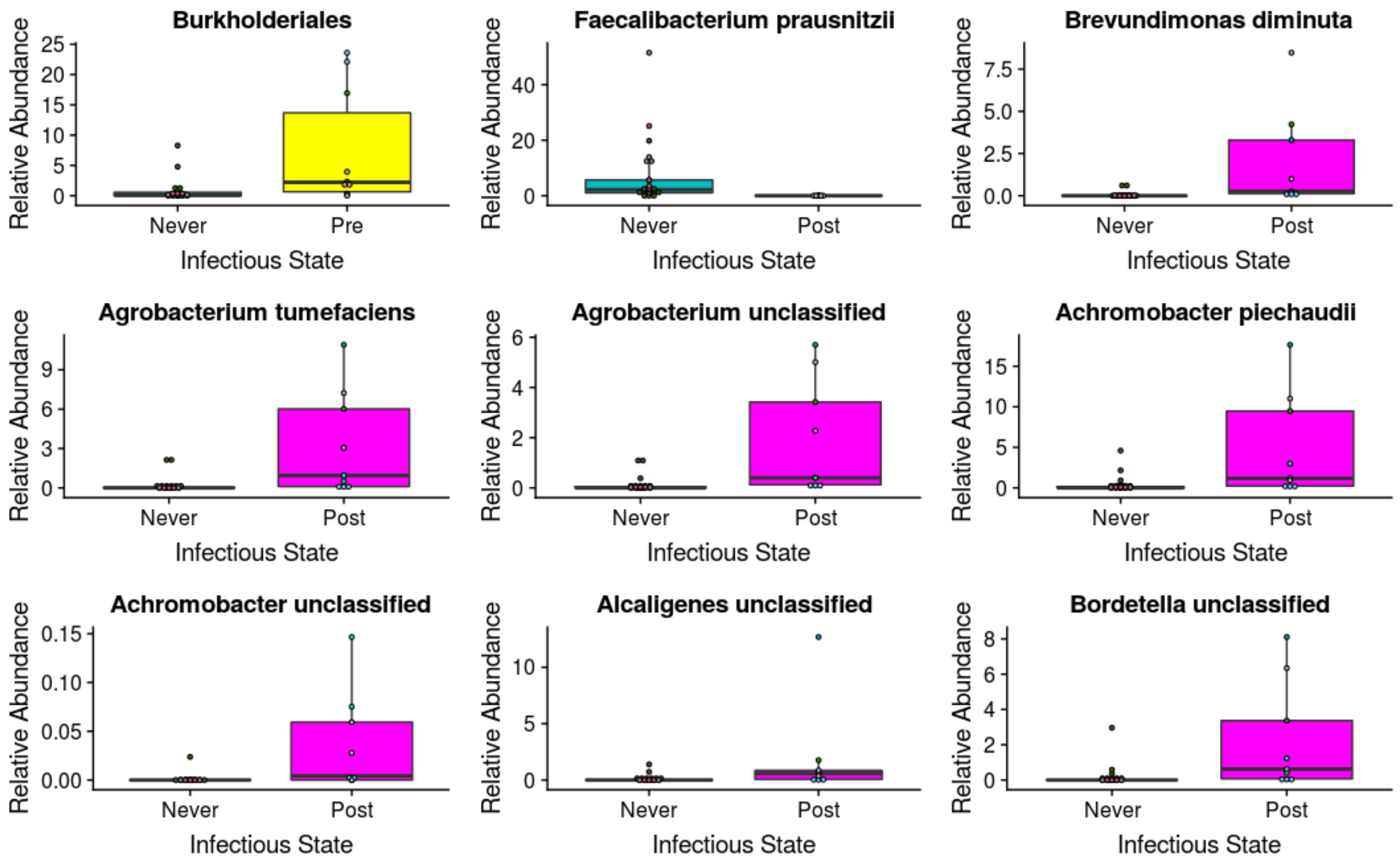

Supplemental Figure 13: **Burkholderiales is significantly different in relative abundance in samples from pre-IC patients whereas multiple species are significantly different in abundance between samples from NIC patients and post-IC patients.**
The order Burkholderiales was found to be significantly increased in relative abundance from pre-IC samples, whereas seven species were determined to be significantly different in relative abundance between samples from NIC and IC patients (Wilcoxon rank sum test with correction for false discovery at an alpha value of 0.05). Points are colored by individual patient (**Sup Fig 1**). This data was generated from metagenomic shotgun sequencing data.

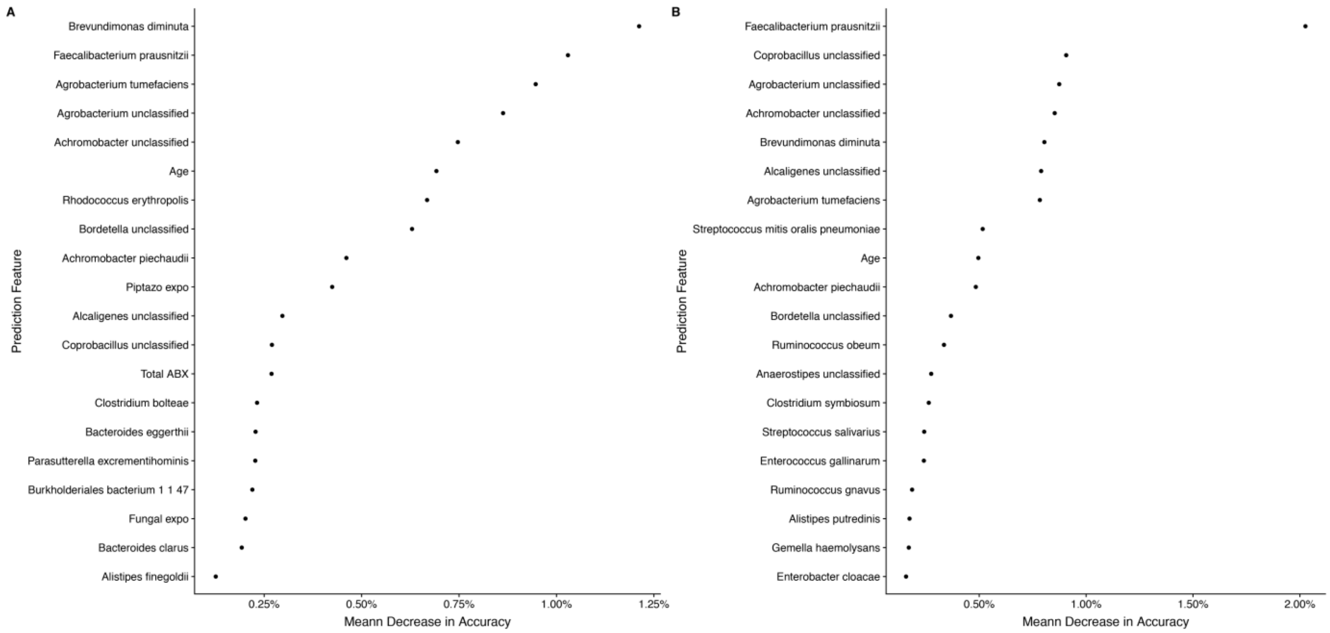

Supplemental Figure 14: **Random Forest Models built on IC samples treated or not treated with vancomycin reveal similar underlying features.**

Two random forest models were built using NIC samples and either IC samples that were not exposed to vancomycin within 2 weeks A) or were exposed to vancomycin within two weeks B). Both species and metadata were used as classification features in the classification of samples either coming from NIC or IC patients. The top 20 most important features are ranked by their mean decrease in accuracy when the feature is randomly permuted after model training. This data was generated from metagenomic shotgun sequencing data.
